# Lipidomic profiling of human adiposomes identifies specific lipid shifts linked to obesity and cardiometabolic risk

**DOI:** 10.1172/jci.insight.191872

**Published:** 2025-06-23

**Authors:** Abeer M. Mahmoud, Imaduddin Mirza, Elsayed Metwally, Mohammed H. Morsy, Giorgia Scichilone, Monica C. Asada, Amro Mostafa, Francesco M. Bianco, Mohamed M. Ali, Mario A. Masrur, Chandra Hassan, Brian T. Layden

**Affiliations:** 1 Department of Medicine, Division of Endocrinology, Diabetes, and Metabolism, College of Medicine, University of Illinois Chicago, Chicago, Illinois, USA.; 2 Department of Kinesiology and Nutrition, College of Applied Health Sciences,; 3 Department of Pharmacology, College of Medicine,; 4 Department of Surgery, College of Medicine, and; 5 Department of Biobehavioral Nursing Science, College of Nursing, University of Illinois Chicago, Chicago, Illinois, USA.; 6 Jesse Brown Veterans Affairs Medical Center, Chicago, Illinois, USA.

**Keywords:** Inflammation, Metabolism, Vascular biology, Adipose tissue, Lipidomics, Obesity

## Abstract

**BACKGROUND:**

Obesity, a growing health concern, often leads to metabolic disturbances, systemic inflammation, and vascular dysfunction. Emerging evidence suggests that adipose tissue-derived extracellular vesicles (adiposomes) may propagate obesity-related complications. However, their lipid composition and effect on cardiometabolic state remain unclear.

**METHODS:**

This study examined the lipid composition of adiposomes in 122 participants (75 in obesity group, 47 in lean group) and its connection to cardiometabolic risk. Adiposomes were isolated via ultracentrifugation and characterized using nanoparticle tracking and comprehensive lipidomic analysis by mass spectrometry. Cardiometabolic assessments included anthropometry, body composition, glucose-insulin homeostasis, lipid profiles, inflammatory markers, and vascular function.

**RESULTS:**

Compared with lean controls, individuals with obesity exhibited elevated adiposome release and shifts in lipid composition, including higher ceramides, free fatty acids, and acylcarnitines, along with reduced levels of phospholipids and sphingomyelins. These alterations strongly correlated with increased BMI, insulin resistance, systemic inflammation, and impaired vascular function. Pathway enrichment analyses highlight dysregulation in glycerophospholipid and sphingolipid metabolism, bile secretion, proinflammatory pathways, and vascular contractility. Machine-learning models utilizing adiposome lipid data accurately classified obesity and predicted cardiometabolic conditions, such as diabetes, hypertension, dyslipidemia, and liver steatosis, achieving accuracy above 85%.

**CONCLUSION:**

Obesity profoundly remodels the adiposome lipid landscape, linking lipid changes to inflammation, metabolic dysfunction, and vascular impairment. These findings underscore adiposome lipids as biomarkers for obesity and related cardiometabolic disorders, supporting personalized interventions and offering therapeutic value in risk stratification and treatment.

**FUNDING:**

This project was supported by NIH grants R01HL161386, R00HL140049, P30DK020595 (PI: AMM), R01DK104927, and P30DK020595 as well as by a VA Merit Award (1I01BX003382, PI: BTL).

## Abstract 2

This study reveals obesity-related changes in adiposome lipids that promote inflammation, metabolic dysfunction, and vascular issues, highlighting new diagnostic and therapeutic possibilities.

## Graphical abstract

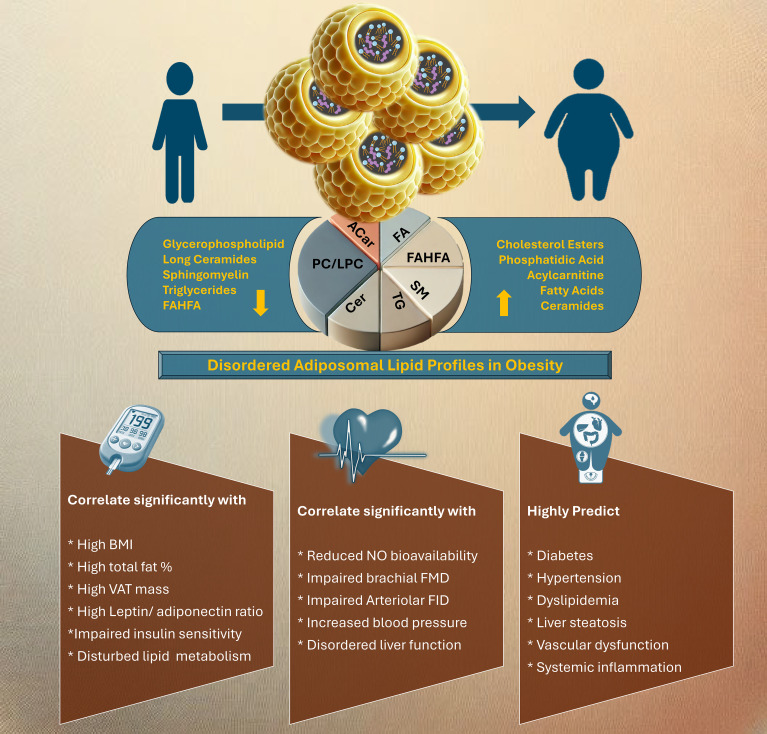

## Introduction

Obesity has become a critical health concern worldwide due to its rapid and sustained rise. According to the most recent data from the CDC, obesity has reached critical levels in the United States (1). This escalating trend correlates with an increased risk of developing a range of chronic disorders, including cardiovascular diseases and metabolic syndrome (2). However, the precise molecular underpinnings by which obesity catalyzes or exacerbates these diseases have not been completely delineated. One well-documented hallmark of obesity is the expansion of adipose tissue, a highly active endocrine organ secreting numerous hormones that regulate critical processes such as metabolism, insulin sensitivity, appetite control, and vascular function (3). In obesity, however, dysfunctional adipose tissue and dysregulated secretome contribute to local and systemic inflammation, insulin resistance, and other metabolic derangements (4–6). Recent findings from in vitro and preclinical studies emphasize the significance of extracellular vesicles (EVs) derived from adipose tissues, currently known as adiposomes, in various metabolic and cardiovascular disturbances (7, 8). However, corresponding findings in human adiposomes and their role in metabolic and vascular dysfunction are lacking.

Over the past decade, EVs have emerged as significant players in metabolic regulation and interorgan communication. Once considered mere byproducts of cellular processes, EVs — encompassing exosomes (~30–150 nm in diameter) and microvesicles (~100–1000 nm) — are now appreciated as sophisticated carriers of lipids, proteins, and nucleic acids (9). Numerous studies initially focused on proteins and miRNAs, identifying cargo that can modulate inflammatory signaling and insulin pathways. However, recent attention has shifted to the lipid composition, given the pivotal role of bioactive lipids in cell signaling, inflammation, and metabolism (10). Among the lipid species commonly identified in EVs are phospholipids and sphingolipids, which serve as key structural components of the vesicle membrane and influence insulin sensitivity and inflammation (11, 12). Growing evidence suggests that lipids are not merely structural elements of EVs but are also functionally active molecules influencing cell signaling and homeostasis (13).

**Figure 1:**
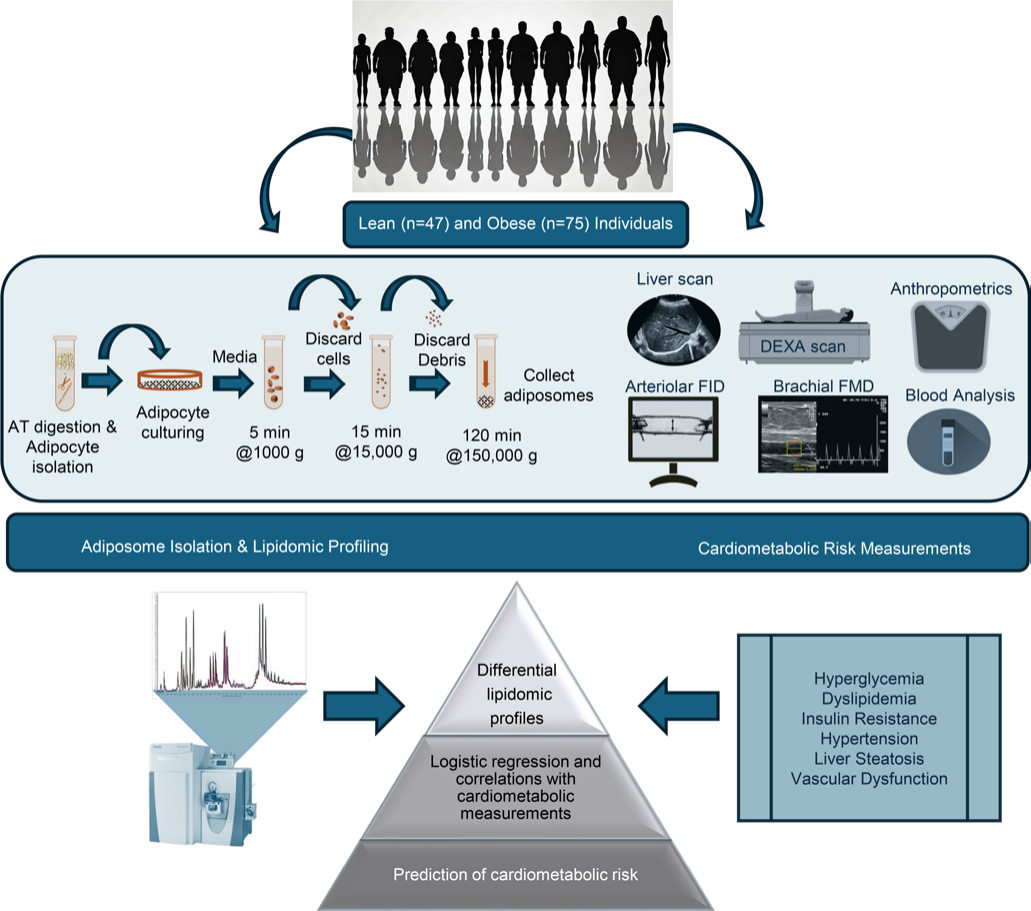
A schematic illustration of the study design comparing individuals from the lean (n = 47) and obese (n = 75) groups.

Adipose tissue releases large quantities of adiposomes that encapsulate numerous bioactive components (14–16). Our previous studies demonstrated that adiposomes isolated from individuals with obesity instigate endothelial dysfunction by impairing caveolae integrity and disrupting nitric oxide (NO) signaling in endothelial cells (17, 18). In a recent pilot study, we observed higher levels of ceramides (Cer) and lower phospholipids levels in adiposomes isolated from participants with obesity compared with lean controls (19). Building on this data, we conducted a comprehensive investigation into the relationship between adiposome lipid profiles and obesity. This study analyzed adiposome lipidomics in a cohort of 122 individuals with obesity and those who are lean, integrating clinical and physiological data and applying machine learning models to elucidate their associations with obesity and related complications, as outlined in Figure 1. Understanding these molecular intricacies holds substantial implications for developing novel therapeutic targets, enhancing diagnostic biomarker discovery, and devising strategies for mitigating obesity-related cardiometabolic complications.

## Results

### Clinical characteristics of the study participants.

All clinical characteristics of the participants with obesity (*n* = 75) and those who are lean (*n* = 47) were summarized in Table 1. The average age for both groups was not statistically different. However, the average BMI was approximately 70% higher in the participants with obesity than the lean controls. Similarly, total fat and visceral fat percentages measured using dual-energy x-ray absorptiometry (DEXA) scanning were 85% and 93% higher, respectively, in participants with obesity than in lean controls. Waist circumference (WC) was significantly higher in the obese group (~38%, *P* < 0.001). Metabolic parameters, including fasting plasma glucose, insulin levels, hemoglobin A1c (HbA1c), and the insulin resistance index, homeostatic model assessment for insulin resistance (HOMA-IR), were significantly higher in individuals with obesity. Lipid profile measurements, including triglycerides, total cholesterol level, and LDL, were markedly elevated in those with obesity compared with lean controls, while the HDL levels were significantly lower in the obese group. Liver function was impaired in the obese group, as evidenced by significant reductions in plasma bilirubin (22%) and total albumin (12%) and a 15% increase in alkaline phosphatase. Liver enzymes, including alanine transaminase (ALT) and aspartate transaminase (AST) tended to be higher in the obese group than in the lean controls, though these differences were not statistically significant. Systemic inflammation biomarkers, such as interleukin 6 (IL-6) and C-reactive protein (CRP), were markedly higher in the obese group. Additional differences included significantly higher leptin (105% higher) and lower adiponectin (84% lower) in individuals with obesity than in lean controls.

Next, we used brachial flow-mediated dilation (FMD), a noninvasive ultrasound technique, to evaluate endothelial function, where higher values suggest better vascular health. Individuals with obesity exhibited a 60% lower brachial FMD than lean controls (*P* < 0.001). Similarly, arteriolar flow-induced dilation (FID), measured ex vivo in adipose tissue-isolated arterioles, was reduced by 45% in participants with obesity compared with lean controls (*P* < 0.001), suggesting impaired peripheral arteriolar vasodilation. Additionally, individuals with obesity show a 48% reduction in NO bioavailability compared with lean controls, indicating impaired endothelial function.

Of the 75 participants with obesity, 34 were hypertensive (~45%), 42 were insulin resistant or had diabetes (56%), 38 were dyslipidemic with disrupted lipid profile (~51%), and 46 had steatotic liver (~61%). Diagnosis of these conditions relied on measurements conducted during study visits and a review of patient medical records. Finally, based on medication information collected from participants, 29 were taking antihypertensive medications (including angiotensin-converting enzyme inhibitors, angiotensin II receptor blockers, β blockers, calcium channel blockers, and diuretics), 28 were on diabetic medications (including metformin, dipeptidyl peptidase-4 inhibitors, glucagon-like peptide-1 agonists, sodium-glucose cotransporter 2 inhibitors, sulfonylureas, or insulin), and 14 participants were on statin therapy.

### Obesity-driven alterations in adiposome lipids.

**Figure 2:**
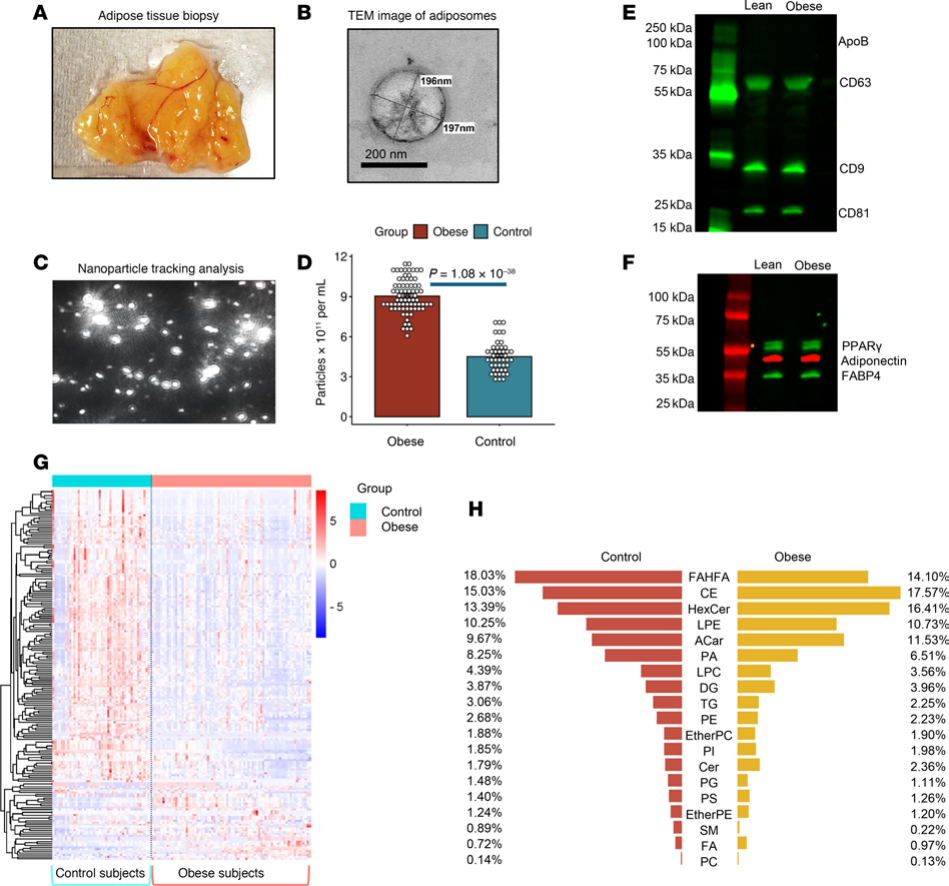
Analysis of adiposomes and lipid alterations in individuals in the obese and lean groups. (**A**) A representative image of the AT biopsy sample. (**B**) TEM image of adiposome particles. Scale bar: 200 nm. (**C**) Nanoparticle tracking analysis displaying adiposome particles in the samples. Original magnification ×20. (**D**) Quantification of adiposome concentration in obese and lean groups (Mann-Whitney U test). (**E** and **F**) Western blot analysis for EV biomarkers and adipocytic proteins. (**G**) Heatmap of adiposome lipid species across lean and obese groups. (**H**) Percentage composition of major lipid classes in the control and obese groups.

To study obesity-driven alterations in adiposome lipids and their role in cardiometabolic dysfunction, human adiposomes were extracted from the visceral adipose tissue (VAT) and characterized by transmission electron microscopy (TEM) and nanoparticle-tracking analysis (NTA) (Figure 2, A–C). These analyses revealed adiposome diameters ranging from 50 to 350 nm. The average concentration of adiposomes was significantly higher in samples from patients with obesity compared with those from lean controls (9.0 × 10¹¹ particles/mL versus 4.5 × 10¹¹ particles/mL; *P* < 0.001) (Figure 2D). Protein analysis of the adiposomes confirmed the presence of conventional EVs biomarkers, including tetraspanins CD9, CD81, and CD63, while showing no evidence of lipoprotein contamination (Apolipoprotein B; APOB) (Figure 2E). Additionally, the adiposomes expressed adipocyte-specific proteins such as PPARγ, adiponectin, and FA-binding protein 4 (FABP4) (Figure 2F), further confirming their adipocytic origin.

**Figure 3:**
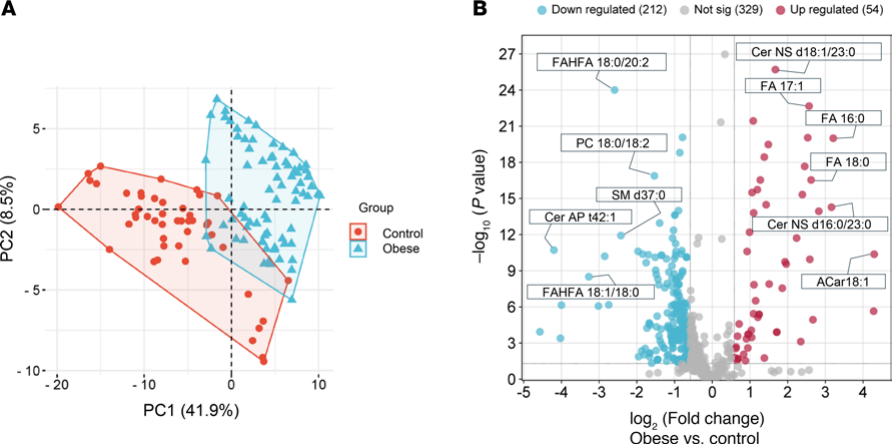
Differential adiposome lipids in individuals in the obese and lean groups. (**A**) Principal Component Analysis (PCA) plot of adiposome lipids in obese and lean groups. (**B**) Volcano plot illustrates the differential lipid expression (log_2_ fold change) between obese and lean groups (–log_10_ *P* value; unpaired 2-tailed *t* test with multiple testing correction; Benjamini-Hochberg FDR).

Comprehensive lipidomics analysis identified 595 lipid species spanning 19 major lipid classes (Figure 2G). Among these, 266 lipids exhibited significant alterations in the adiposomes of participants with obesity compared with lean controls, with 54 upregulated and 212 downregulated (FDR < 0.05) after adjusting for age, sex, and race/ethnicity. Comparing the percentage composition of lipid classes (Figure 2H), the most considerably elevated lipids in the obese group belonged to the classes of FA, acylcarnitines (Acar), Cer, hexosyl ceramides (HexCer), cholesterol esters (CE), and diacylglycerols (DG). In contrast, lipids considerably enriched in the control group included phospholipids (phosphatidylcholine [PC]), phosphatidylethanolamine (PE), lysophosphatidylcholine (LPC), phosphatidic acid (PA), triglycerides (TG), SM, and fatty acyl esters of hydroxy FA (FAHFA). Principal Component Analysis (PCA) revealed a clear separation of the samples into 2 distinct groups in the PCA plot (Figure 3A). The volcano plot analysis was then used to visualize the differentially abundant lipids in the isolated adiposomes from participants who are lean and those with obesity (Figure 3B). Notably, Cer-NS d18:1/23:0 exhibited a significant increase, while FAHFA 18:0/20:2 demonstrated a significant decrease in the obese group compared with the control group.

**Figure 4:**
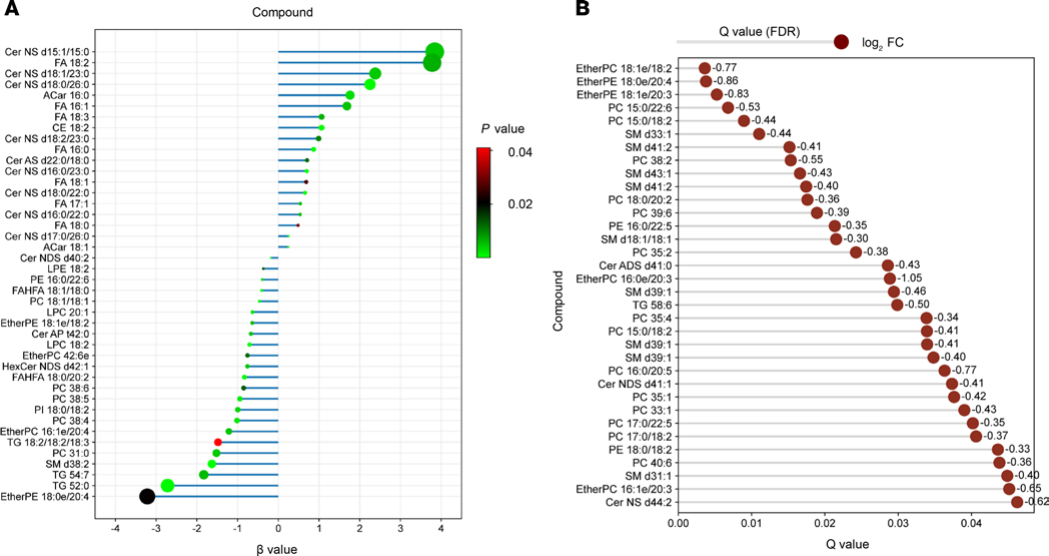
Regression analysis and association of adiposome lipids with obesity status. (**A**) Regression plot showing the association between lipid species and obesity status, arranged by their β values (linear regression) and their associated *P* values. (**B**) Horizontal lollipop chart shows the differential lipid abundance from class 2 and 3 individuals with obesity using log_2_ fold change to represent the magnitude of change. For statistical significance, an unpaired 2-tailed *t* test with multiple testing correction (Benjamini-Hochberg FDR) was used.

To assess whether specific lipid species were associated with BMI in this population, we conducted a linear regression analysis (Figure 4A). Nineteen lipid species, primarily in the Cer, FA, CE, and Acar lipid classes, demonstrated positive β values, indicating a positive correlation with BMI. Conversely, negative β values were found for 23 lipid species, predominantly from phospholipid, sphingomyelin (SM), FAHFA, and TG lipid classes, suggesting an inverse relationship with the BMI. To evaluate the model’s goodness of fit, we used 3 key metrics: adjusted R-squared, root mean squared error (RMSE), and the *P* value of the regression model. The adjusted R-squared value was 0.85, indicating that the adiposome lipidomics data explained 85% of the variability in BMI.

The RMSE value of 4.9 represents the average error magnitude between the observed BMI values and those predicted by the regression model. The *P* value for the overall regression model was found to be 5.47 × 10^–6^, indicating that the independent variables (lipid species) included in the model are valid predictors of BMI. In summary, the linear regression analysis identified 42 specific lipid species associated with changes in BMI, providing valuable insights into the relationship between adiposome lipid composition and BMI.

Furthermore, our results also identified a group of 34 lipids, predominantly of the phospholipid and SM lipid categories, that were significantly less abundant in adiposomes from individuals with class 3 obesity (BMI ≥ 40 kg/m^2^) compared with those from class 2 (BMI, 35–39.9 kg/m^2^). This difference remained significant after adjusting for potential confounding variables such as age, sex, and race/ethnicity (Figure 4B; FDR < 0.05). This differential human lipid profile may have important implications for understanding the functional differences in adipose tissue biology and the associated metabolic dysregulation in higher BMI categories.

### Unique adiposome lipid signatures differentiate individuals with obesity and those who are lean.

**Figure 5:**
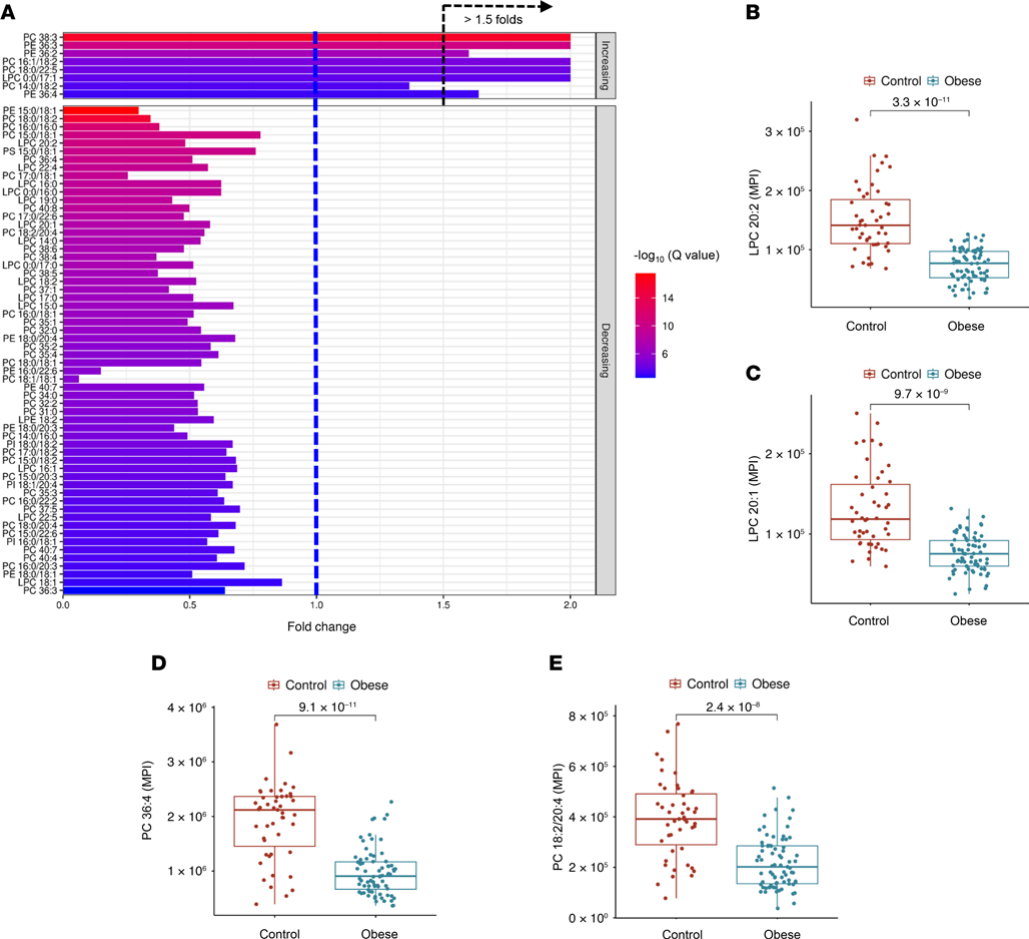
Differential phospholipid profiles in adiposomes of individuals in the obese and lean groups. (**A**) Bar plot of fold changes in the top 69 phospholipid and lysophospholipid species between obese and control (lean) groups and their significance (–log_10_ *q* values). (**B**–**E**) Box plots showing examples of the significantly altered phospholipid species between the obese and lean groups. For statistical significance, an unpaired 2-tailed *t* test with multiple testing correction (Benjamini-Hochberg FDR) was used. MPI, mean peak intensity.

**Figure 6:**
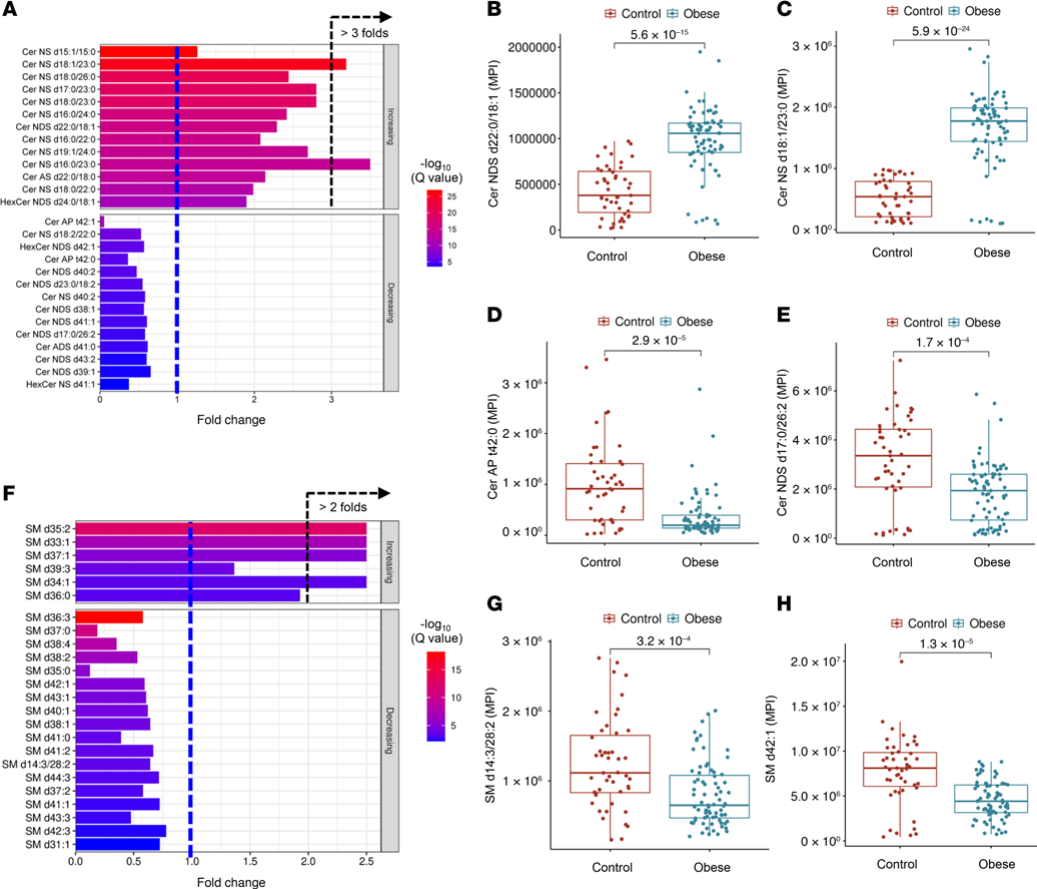
Ceramide and sphingomyelin alterations in adiposomes of individuals in the obese and lean groups. (**A**) Bar plot of fold changes in Cer and HexCer between obese and control groups and their significance (–log_10_ *q* values). (**B**–**E**) Box plots of key Cer showing significant differences between groups. (**F**) Bar plot of fold changes in the expression levels of SM in obese and lean groups. (**G** and **H**) Box plots of selected SM species show significant differences between the control and obese groups. For statistical significance, an unpaired 2-tailed *t* test with multiple testing correction (Benjamini-Hochberg FDR) was used.

In this study, adiposome lipid profiling revealed marked differences in various lipid species between individuals with obesity and lean healthy controls. Notably, LPCs and PCs, key phospholipid components, were significantly lower in adiposomes from individuals with obesity, indicating an overall reduction in phospholipid species. For instance, LPC 20:1, LPC 20:2, PC 36:4, and PC 18:2/20:4 all displayed significantly lower levels (*P* < 0.01). Ether-linked phospholipids (EtherPE 18:0e/20:4 and EtherPC 16:0e/20:4) were enriched in lean participants, further underscoring disrupted phospholipid metabolism in obesity (Figure 5, A–E). Ceramides, SM, TG, ACar, FA, and FAHFAs also showed differential abundance. Ceramides exhibited notable shifts: certain species (e.g., Cer NDS d22:0/18:1) were significantly higher in individuals with obesity, while lean participants showed higher levels of others (e.g., Cer AP t42:0) (Figure 6, A–E). Interestingly, elevated Cer in lean individuals often contained more carbons and unsaturated bonds. SM likewise differed, with SM d14:3/28:2 and SM d42:1 enriched in lean participants, whereas SM d33:1 and SM d39:3 were higher in the obese group (Figure 6, F–H). Overall, total Cer levels were significantly higher in obesity, while total SM levels were lower, suggesting enhanced SM hydrolysis of Cer, a process known to drive inflammation and insulin resistance (20).

**Figure 7:**
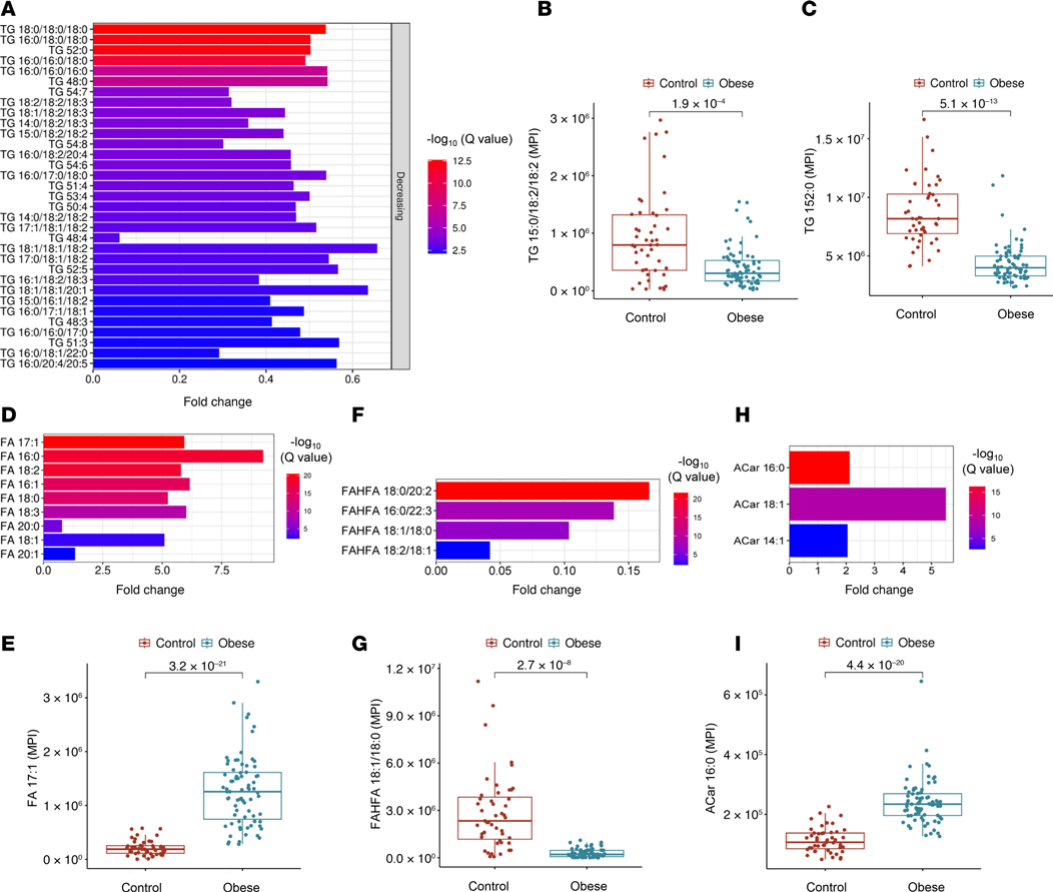
Differential profiles of triglycerides, fatty acids, FAHFAs, and acylcarnitines. (**A**, **D**, **F**, and **H**) Bar plot of fold changes in TG species (**A**), fatty acid species (**D**), FAHFAs (**F**), and ACar species (**H**) between obese and control groups and their significance (–log_10_ *q* values [FDR]). (**B**, **C**, **E**, **G**, and **I**) Box plots illustrate altered TG species (**B** and **C**), FA 17:1 (**E**), FAHFA 18:1/18:0 (**G**), and ACar 16:0 (**I**) in the lean and obese groups. For statistical significance, an unpaired 2-tailed *t* test with multiple testing correction (Benjamini-Hochberg FDR) was used.

TGs were generally more abundant in lean individuals. Specific TGs (e.g., TG 15:0/18:2/18:2 and TG 52:0) were depleted in participants with obesity, suggesting possible impairments in TG synthesis or elevated lipolysis (Figure 7, A–C). Concurrently, higher levels of free FA in the obese group indicated reduced TG storage and increased FA intermediates. Most measured FAs, including FA 16:0, FA 17:1, and FA 18:0, were more abundant in obesity (Figure 7, D and E). FAHFAs, which possess antiinflammatory and insulin-sensitizing properties (21), were lower in individuals with obesity (e.g., FAHFA 18:1/18:0 and FAHFA 16:0/22:3) (Figure 7, F and G). This reduction may reflect diminished protective lipid species in obesity. Conversely, ACar, which are critical for transporting FAs into mitochondria for β-oxidation, were significantly elevated in participants with obesity (e.g., ACar 16:0 and ACar 18:1), suggesting perturbations in lipid metabolism, mitochondrial function, and inflammation (Figure 7, H and I).

Overall, these findings reveal significant obesity-related alterations in the adiposome lipid landscape, including increased levels of Cer, free FAs, and ACar, coupled with lower phospholipids, SM, certain TGs, and FAHFAs. These lipidomic alterations indicate a substantial metabolic remodeling of adiposome function, likely contributing to inflammation, insulin resistance, and other obesity-related dysfunctions. Understanding these lipidomic changes offers insights into obesity pathophysiology and points to potential therapeutic targets to restore healthy lipid balance.

### Sex and racial/ethnic effects on adiposome lipidomics in individuals with obesity.

To study the effect of sex on adiposome lipid profiles, the current study included 45 female participants with obesity and 30 male participants with obesity. Among clinical measures, the total fat percentage was higher in females (53.1 ± 6.4) than in males (46.8 ± 6.9; *P* = 0.02), while hemoglobin was lower in females (12.3 ± 1.2) compared with males (13.5 ± 0.8; *P* = 0.0008). Adiposome lipidomics; certain lipid species, including SM d41:1, PI 18:1/18:2; and PC 19:0 lipid species were higher in females (log_2_ fold change [FC] = 1.4, 1.3, 1.1), though these differences showed only borderline significance after multiple-testing correction. Participants represented 3 racial/ethnic groups: African American (AA, *n* = 42), Hispanic (H, *n* = 20), and Non-Hispanic White (NHW, *n* = 13). No significant differences were observed in clinical or physiological measures across these groups. However, after correction for multiple testing, 18 adiposome lipid species showed significant differences, primarily between AA and the other 2 groups. Phospholipids, including PC, PE, and ether PC, were lower in AA than H and NHW, while PA were consistently higher in AA and H than in NHW. Two sphingomyelin (SM) species were lower in AA versus NHW; SM d37:1 also differed between AA and H, and SM d39:1 differed between H and NHW. Two Cer species (Cer-NDS d18:0/16:0 and Cer-NS d18:1/26:0) were significantly higher in AA and H than in NHW. Three FAs (FA 16:1, FA 18:1, and FA 18:0) were also higher in AA. However, total Cer and SM levels did not differ significantly among the 3 groups (Table 2).

### Adiposome lipidomic differences across individuals with diabetes, hypertension, and dyslipidemia.

**Figure 8:**
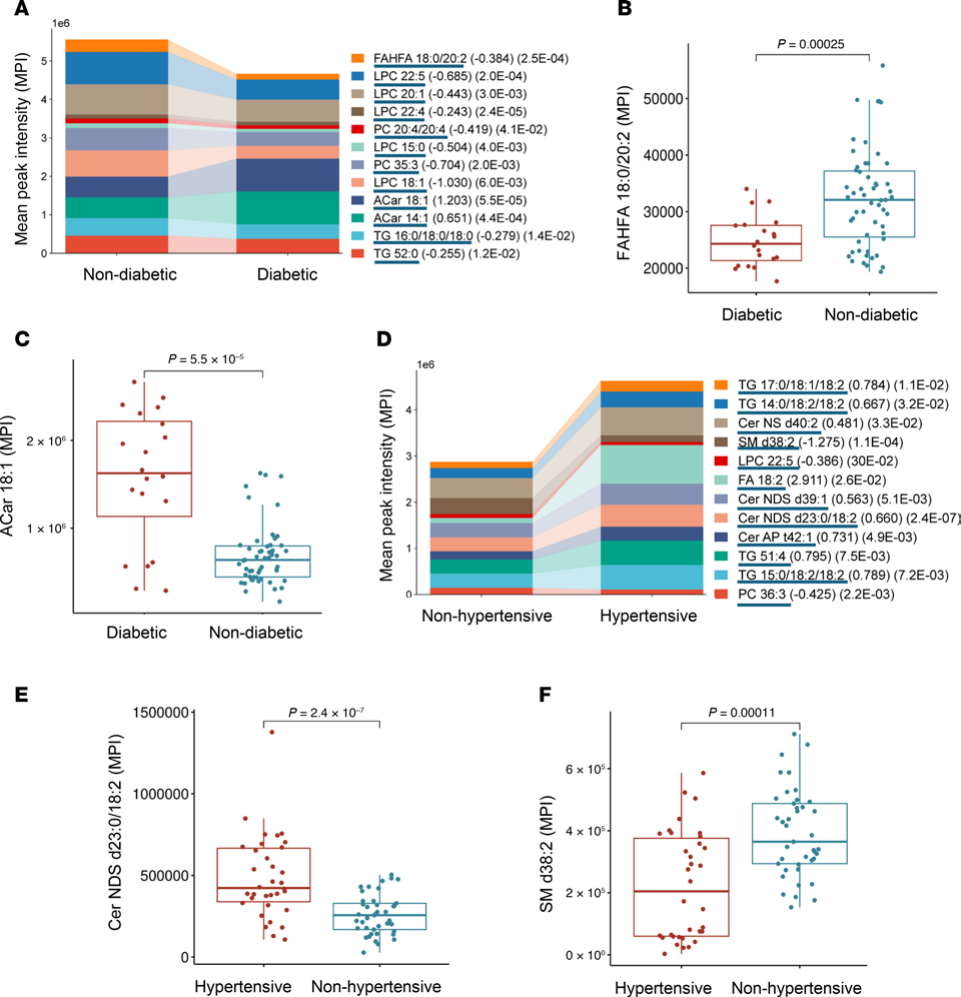
Lipidomic alterations in diabetic and hypertensive individuals. (**A** and **D**) Stacked bar plots show the distribution of lipid classes (log_2_ FC and FDR values are provided in parentheses) in individuals with diabetes versus those without diabetes (**A**) and those who are hypertensive versus those who are nonhypertensive (**D**). (**B**, **C**, **E**, and **F**) Boxplots for lipids altered between diabetic versus nondiabetic groups (**B** and **C**) and hypertensive versus nonhypertensive groups (**E** and **F**). For statistical significance, an unpaired 2-tailed *t* test with multiple testing correction (Benjamini-Hochberg FDR) was used.

**Figure 9:**
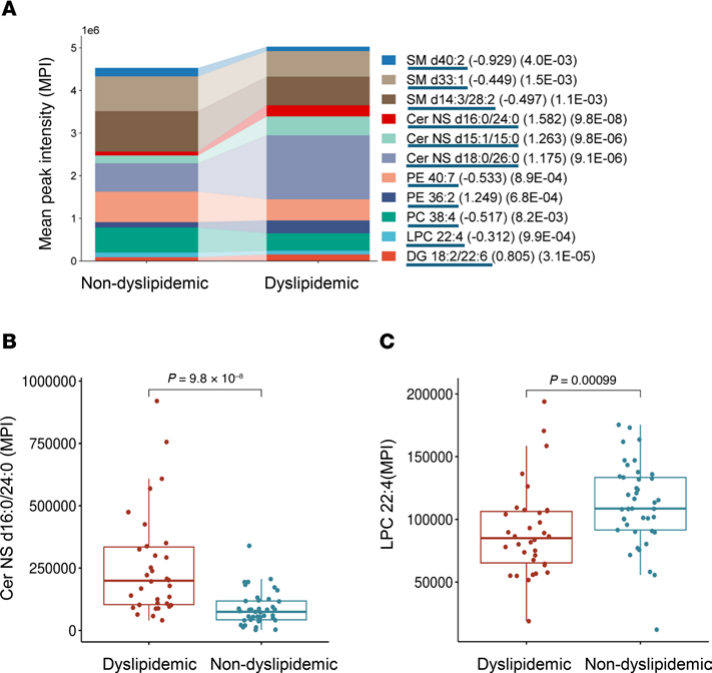
Lipidomic alterations in dyslipidemic individuals. (**A**) Stacked bar plots show the distribution of lipid classes (log_2_ FC and FDR values in parentheses) in dyslipidemic versus nondyslipidemic individuals. (**B** and **C**) Box plots for lipids altered between dyslipidemic and nondyslipidemic groups. For statistical significance, an unpaired 2-tailed *t* test with multiple testing correction (Benjamini-Hochberg FDR) was used.

Our data reveal differential abundances of specific lipid species in diabetic, hypertensive, and dyslipidemic participants with obesity compared with metabolically healthy counterparts with obesity. Individuals with diabetes showed lower levels of FAHFA 18:0/22:0 (*P* = 0.00025) and several phospholipid and TG species, whereas ACar (ACar 18:1 and 16:1) were significantly higher in participants with diabetes than in those without it (Figure 8, A–C). This shift toward increased ACar levels may reflect altered FA oxidation or mitochondrial dysfunction in diabetes. Adiposome lipid profiles also differed between obese individuals with and without hypertension. Cer-NDS d23:0/18:2 and other Cer were elevated in the hypertensive group, while SM d38:2 was significantly lower, suggesting a shift toward Cer production that could promote inflammation and vascular dysfunction. Linoleic acid (FA 18:2) and specific TGs (TG 51:4, TG 15:0/18:2/18:2, TG 17:0/18:1/18:2) were also higher in the hypertensive group, possibly reflecting altered lipid metabolism or impaired lipid storage (Figure 8, D–F). In dyslipidemic versus nondyslipidemic individuals with obesity, there was a substantial increase in Cer-NDS d16:0/24:0 (*P* = 9.8 × 10^–8^) and DG 18:2/22:6, paired with reduced levels of phospholipids, such as phosphatidylserine (PS 15:0/18:1) and SM (SM d40:2, SM d33:1) (Figure 9, A–C). This pattern indicates a lipid imbalance favoring Cer and DG accumulation, potentially exacerbating cardiometabolic risk.

Medications also contributed to these adiposome lipidomic differences. Among 12 patients who are hypertensive treated with calcium channel blockers (amlodipine, nifedipine, verapamil) alone or in combination with β blockers (carvedilol, metoprolol), adiposomes contained significantly higher TG 17:0/18:0/18:1 (log_2_ FC = 3.28; *P* = 0.0004) and SM d37:1 (log_2_ FC = 2.06; *P* = 0.007) than in other individuals with obesity. These patients further displayed elevated SM d34:2, TG 56:8, and TG 16:0/18:2/22:6 relative to hypertensive patients not receiving these medications. Moreover, statin therapy was associated with lower FA 18:1 levels (log_2_ FC = –2.72; *P* = 0.0006). In contrast, diabetic medications did not produce notable changes in adiposome lipid profiles, and the single participant receiving omega-3 supplementation did not provide sufficient data for analysis. These findings illustrate that comorbidities like diabetes, hypertension, and dyslipidemia correspond to distinct adiposome lipid signatures and show that certain medications further modify these profiles. Such insights underscore the need for individualized therapeutic strategies in managing obesity-related cardiometabolic disorders.

### Adiposome lipid signature is differentially associated with cardiometabolic risk.

**Figure 10:**
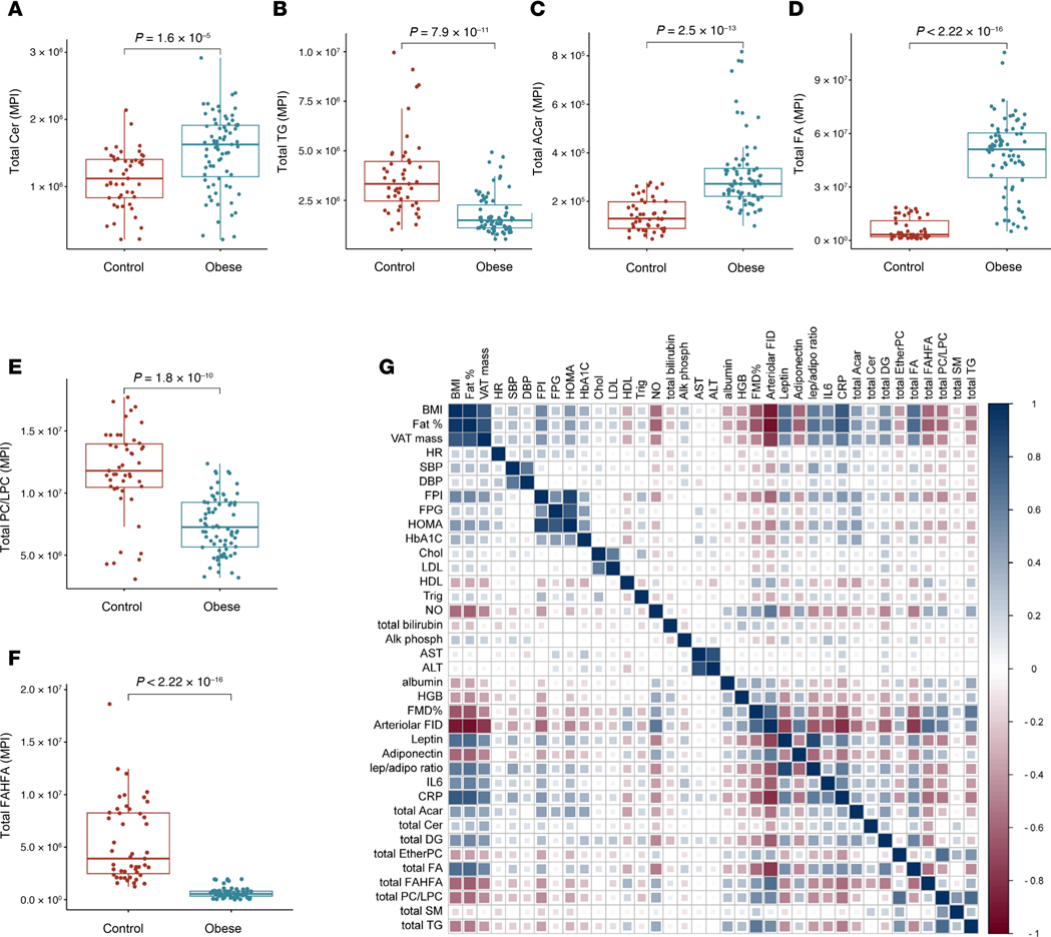
Total lipid classes and correlation with clinical parameters. (**A**–**F**) Box plots comparing total lipid class between obese and lean groups. (**G**) Correlation heatmap displaying the relationships between lipid classes and clinical parameters in individuals in the obese and lean groups. For statistical significance, an unpaired 2-tailed *t* test with multiple testing correction (Benjamini-Hochberg FDR) was used.

A total of 186 adiposome lipids showed significant associations (FDR < 0.05) with cardiometabolic risk features after adjusting for age, sex, and race/ethnicity. These included 100 phospholipids, 28 TGs, 23 Cers, 19 SMs, 11 FAs and FAHFAs, 3 ACar, and 2 DG. Lipids were summed up within each class to examine their relationship to cardiometabolic risk factors, and regression analyses were performed. Total Cer, ACar, and FAs were elevated in participants with obesity compared with lean controls, whereas total TGs, phospholipids (PC/LPC), and FAHFAs were lower in those with obesity (Figure 10, A–F). Regression results indicated that total Cer, ACar, DG, and FA were strongly linked to BMI, fat percentage, glucose-lipid metabolism, vascular function, liver function, and systemic inflammation. Measures of adiposity (BMI, fat percentage, VAT mass) showed positive correlations with proinflammatory lipids such as ACar (*r* > 0.4, *P* < 0.001) and Cer (*r* > 0.5, *P* < 0.01) while correlating negatively with phospholipids (*r* < –0.5, *P* < 0.001) and FAHFAs (*r* < –0.5, *P* < 0.001). HOMA-IR, an indicator of insulin resistance, was positively associated with Cer and free FAs but negatively correlated with phospholipids. IL-6 and CRP similarly correlated positively with Cer, ACar, and FAs while showing negative relationships with FAHFAs, highlighting the potential involvement of these lipids in the inflammation process (Figure 10G).

**Figure 11:**
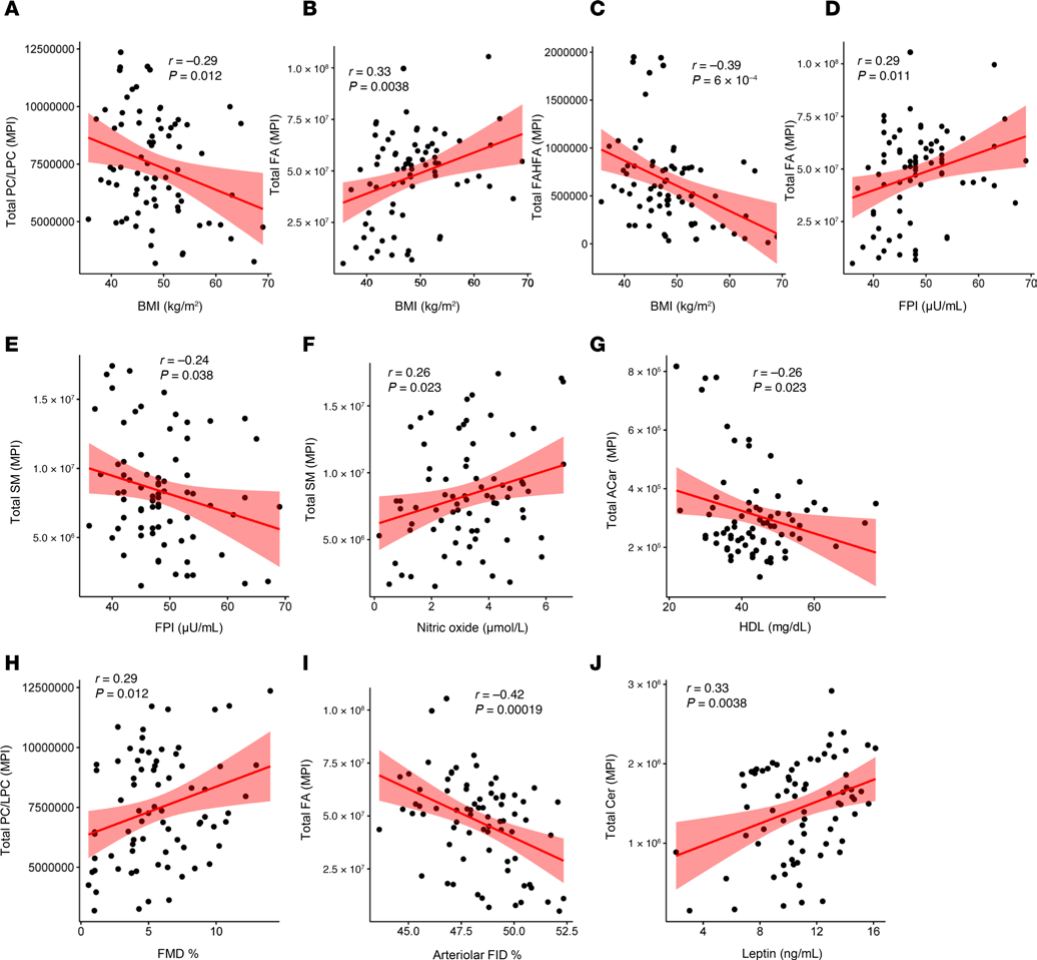
Correlation of lipidomic profiles with clinical parameters in participants with obesity. (**A**–**J**) Scatter plots of lipid levels with cardiometabolic markers in the obese group. Pearson correlation test was used.

Within the obese group, PC, Cer, and FAs were significantly associated with BMI, fasting insulin, leptin, HDL, and arterial vasoreactivity (Figure 11, A–J). Leptin correlated positively with Cer, DG, and FAs but negatively with EtherPC, FAHFAs, and PC/LPC. Adiponectin was positively linked to FAHFAs and SM yet negatively correlated with Cer, underscoring its protective metabolic role. Regarding vascular health, both FMD and arteriolar FID positively correlated with FAHFAs and PC/LPC but were negatively related to Cer, FAs, and ACar. Importantly, lipids found to be less abundant in adiposomes from patients with obesity, such as FAHFAs, SM, and EtherPC, tended to correlate inversely with adiposity and inflammation markers, suggesting that their depletion in obesity exacerbates metabolic dysfunction (Supplemental Tables 1 and 2; supplemental material available online with this article; https://doi.org/10.1172/jci.insight.191872DS1). Overall, the data indicate a striking shift toward proinflammatory lipid profiles alongside diminished protective lipids, linking these changes to obesity-related inflammation, insulin resistance, and vascular impairments.

### Machine learning identifies adiposome lipids as key indicators of cardiometabolic diseases.

**Figure 12:**
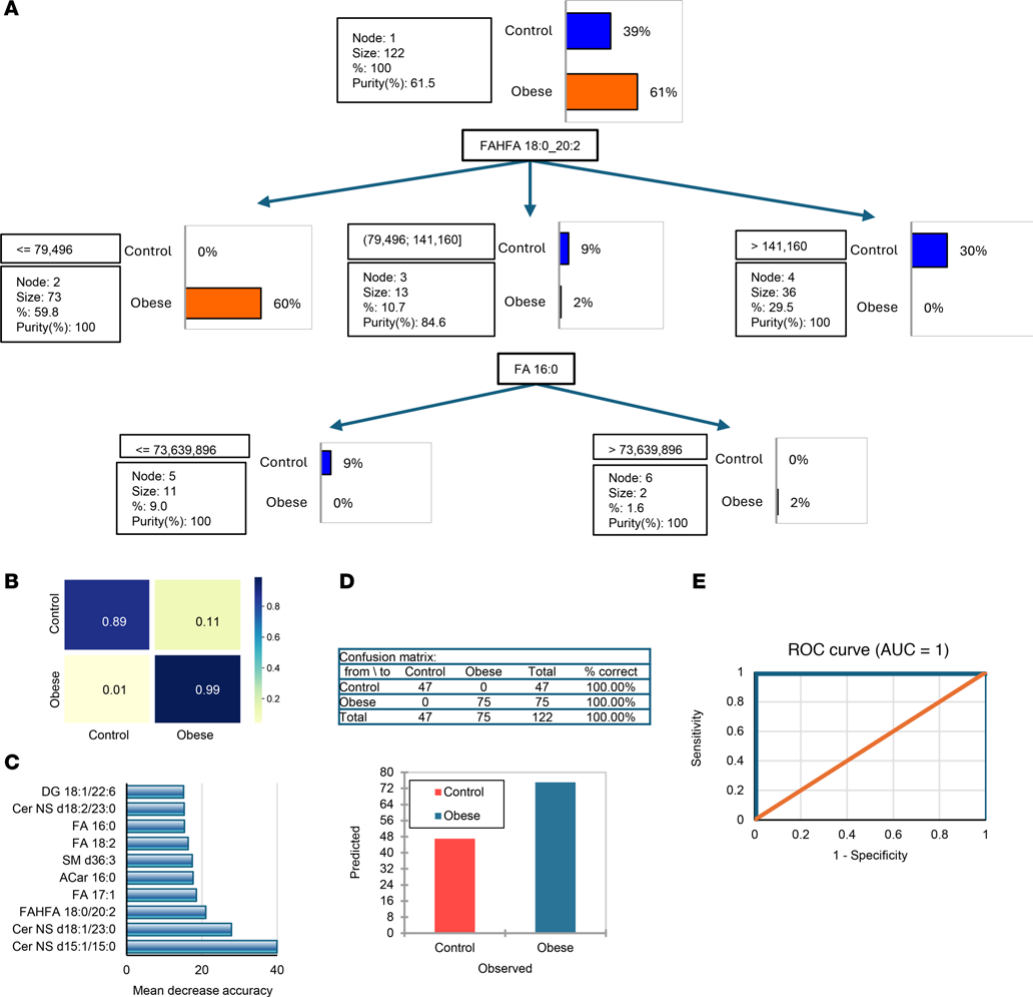
Predictive modeling of obesity status using lipidomic data. (**A**) A decision tree. Visualization highlights key lipids separating obese from lean individuals. (**B**) Random forest confusion matrices display predictive performance for obesity. (**C**) Feature importance plots rank the contribution of individual lipid species for these classifications. (**D**) Corresponding table and bar plot of classification accuracy for obesity. (**E**) ROC curves demonstrate model performance for obesity. Model performance was evaluated using accuracy, sensitivity, specificity, and ROC AUC.

Next, we leveraged these observed differential lipid profiles to develop predictive models (decision tree and random forest) to identify cardiometabolic diseases among individuals with obesity, including diabetes, hypertension, dyslipidemia, and hepatic steatosis, as well as risk factors such as systemic inflammation and vascular dysfunction. First, we used these predictive models to distinguish between control and obese groups based on adiposome lipidomic features. The decision tree model achieved 100% accuracy in predicting obese versus control subjects depending on key lipidomic features (FAHFA 18:0/20:2 and FA 16:0; Figure 12A). The confusion matrix from the random forest model highlights perfect classification accuracy, with 99% of obese and 89% of control samples correctly identified (Figure 12B). The most critical lipids contributing to the model’s accuracy include Cer NS d15:1/15:0, Cer NS d18:1/23:0, FAHFA 18:0/20:2, and FA 17:1 (Figure 12C). These findings demonstrate that these lipidomic markers are highly effective in accurately classifying the samples based on obesity status, as evidenced by the confusion matrix/chart and the receiver operating characteristic (ROC) curve (Figure 12, D and E). Overall, the results reflect the importance of adiposome lipidomic profiling in differentiating between control and obese groups with high accuracy.

**Figure 13:**
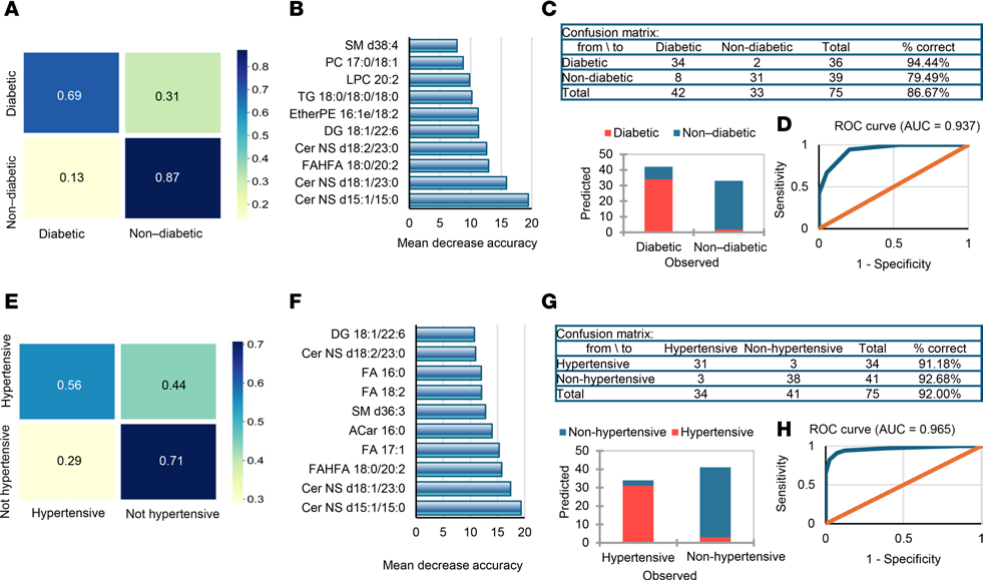
Predictive modeling of diabetes and hypertension. (**A** and **E**) Random forest confusion matrices display predictive performance for diabetes (**A**) and hypertension (**E**). (**B** and **F**) Feature importance plots rank the contribution of individual lipid species for these classifications. (**C** and **G**) Corresponding tables and bar plots of classification accuracy for diabetes (**C**) and hypertension (**G**). (**D** and **H**) ROC curves demonstrate model performance for diabetes (**D**) and hypertension (**H**). Model performance was evaluated using accuracy, sensitivity, specificity, and ROC AUC.

Adiposome lipidomic profiles effectively differentiate diabetic from nondiabetic and hypertensive from nonhypertensive individuals with obesity. In diabetes classification, a random forest model correctly classified 63% of diabetic and 88% of nondiabetic individuals, with Cer-NS d15:1/15:0, Cer-NS d18:1/23:0, and FAHFA 18:0/20:2 emerging as leading predictors alongside phosphocholines, CE, DG, and TG. A decision tree approach performed even better, correctly identifying 94% of diabetic and 79% of nondiabetic participants, yielding an overall accuracy of approximately 87% (Figure 13, A–C). For hypertension, the random forest model achieved 53% and 73% accuracy for hypertensive and nonhypertensive groups, respectively, while the decision tree’s accuracy reached 92%. Top contributors in distinguishing hypertensive versus nonhypertensive individuals included Cer-NS d15:1/15:0, Cer-NS d18:1/23:0, and FAHFA 18:0/20:2, underscoring the importance of Cer and FAHFAs (Figure 13, E–G). ROC curves further illustrated robust discriminative power for both diabetes and hypertension (Figure 13, D and H).

**Figure 14:**
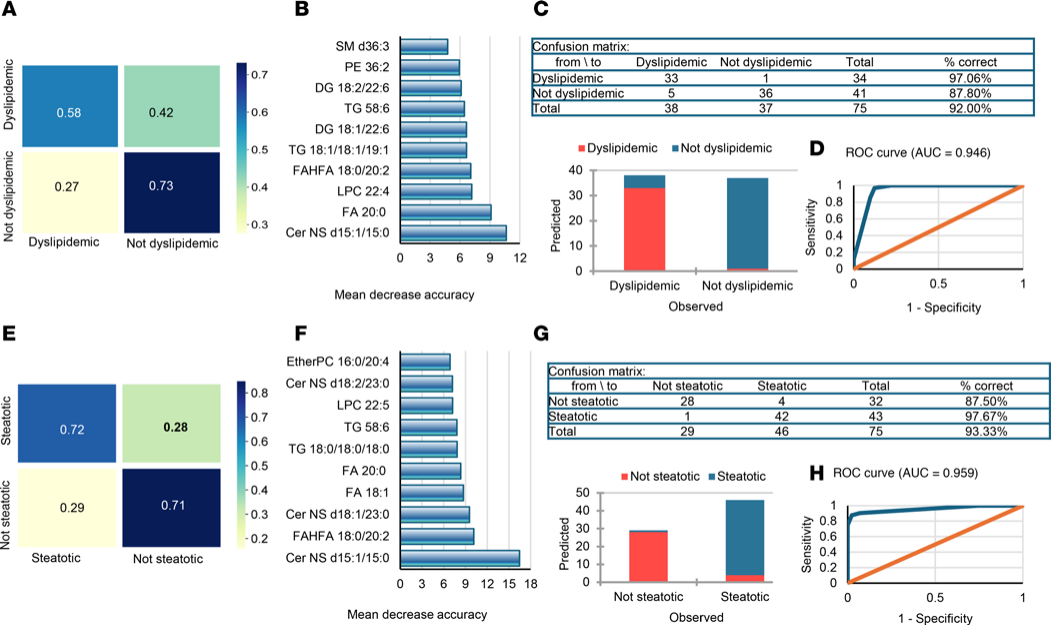
Predictive modeling of dyslipidemia and steatosis. (**A** and **E**) Random forest confusion matrices display predictive performance for dyslipidemia (**A**) and steatosis (**E**). (**B** and **F**) Feature importance plots rank the contribution of individual lipid species for these classifications. (**C** and **G**) Corresponding tables and bar plots of classification accuracy for dyslipidemia (**C**) and steatosis (**G**). (**D** and **H**) ROC curves demonstrate model performance for dyslipidemia (**D**) and steatosis (**H**). Model performance was evaluated using accuracy, sensitivity, specificity, and ROC AUC.

Adiposome lipids also distinguished dyslipidemic from nondyslipidemic individuals with obesity: the random forest model correctly classified 56% of dyslipidemic and 71% of nondyslipidemic individuals, whereas the decision tree model accurately identified 97% and 88%, respectively, yielding an overall accuracy of 92%. Cer NS d15:1/15:0, FA 20:0, and LPC 22:4 lipid species were among the key lipid predictors (Figure 14, A–D). For liver steatosis, random forest correctly classified 72% of steatotic and 71% of nonsteatotic individuals, while the decision tree model achieved ~88% and ~98% accuracy in these groups, respectively. Cer-NS d15:1/15:0, FAHFA 18:0/20:2, and Cer-NS d18:1/23:0 emerged as the top contributing lipids (Figure 14, E–H).

**Figure 15:**
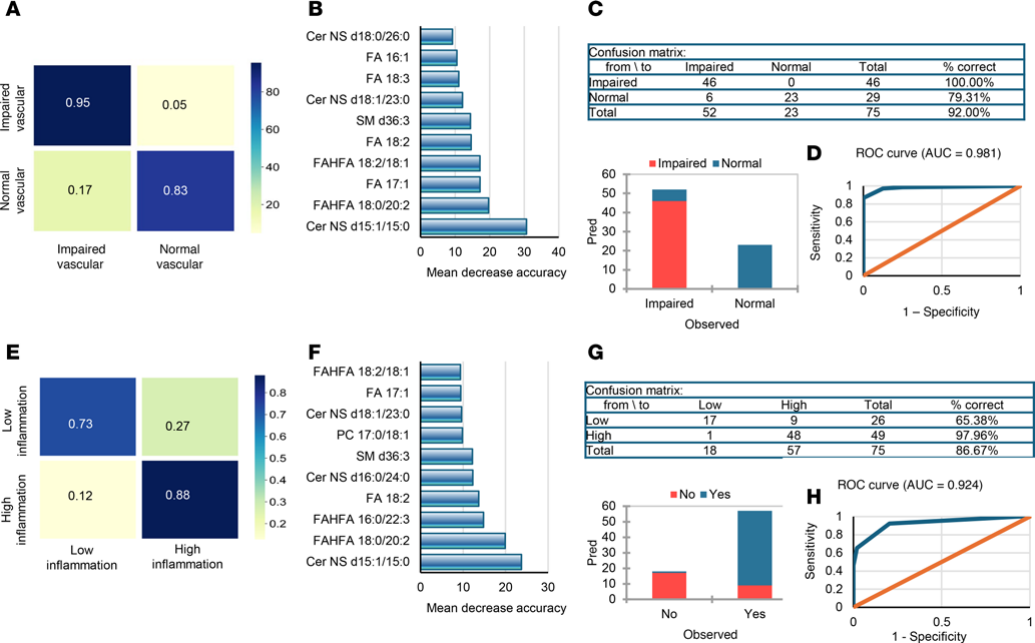
Predictive modeling of vascular dysfunction and inflammation. (**A** and **E**) Random forest confusion matrices display predictive performance for vascular dysfunction (**A**) and inflammation (**E**). (**B** and **F**) Feature importance plots rank the contribution of individual lipid species for these classifications. (**C** and **G**) Corresponding tables and bar plots of classification accuracy for vascular dysfunction (**C**) and inflammation (**G**). (**D** and **H**) ROC curves demonstrate model performance for vascular dysfunction (**D**) and inflammation (**H**). Model performance was evaluated using accuracy, sensitivity, specificity, and ROC AUC.

Individuals with obesity were classified as having vascular dysfunction if their brachial FMD or arteriolar FID fell below the average values of lean, healthy controls (6.8% for brachial FMD and 76.1% for arteriolar FID). A random forest model accurately predicted 98% of those with impaired vascular function and 89% with normal function. Its top predictors included Cer-NS d15:1/15:0, FAHFA 18:0/20:2, and FA 17:1. A decision tree model achieved 100% accuracy in impaired function and 79% in normal function, with an AUC of 0.98 and overall accuracy of 92% (Figure 15, A–D). Individuals with obesity were also classified as having high inflammation if their circulating levels of IL-6 and CRP were higher than the average in the lean, healthy controls (5.4 pg/mL for IL-6 and 0.67 mg/dL for CRP). The random forest model correctly predicted 73% of those with low inflammation and 89% with high inflammation. Its top predictors were Cer-NS d15:1/15:0, FAHFA 18:0/20:2, and FAHFA 16:0/22:3. The decision tree model accurately classified 65% of individuals with obesity with low inflammation and 98% of those with high inflammation, yielding an AUC of 0.92 and an overall accuracy of 86.7% (Figure 15, E–H). Collectively, these results underscore the value of adiposome lipid profiling combined with machine-learning algorithms in classifying obesity-related comorbidities and highlight the potential of adiposome lipidomics as a powerful tool for identifying metabolic dysfunction and guiding personalized interventions in individuals with obesity.

### Pathway enrichment analysis.

**Figure 16:**
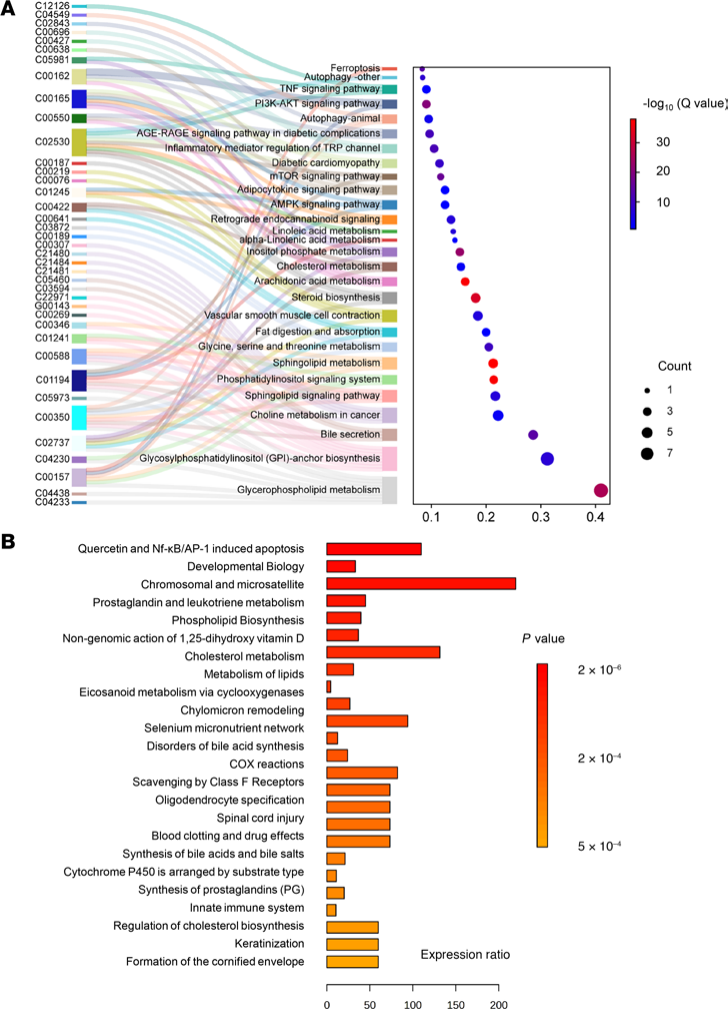
Pathway enrichment analysis of differential adiposome lipid species. (**A**) A Sankey plot visualizes the connection between lipids (labeled with KEGG codes) and enriched KEGG pathways. Each molecule is linked to its associated pathways, with the statistical significance shown in the bubble plot. (**B**) Bar chart of KEGG enrichment analysis showing the top biological processes related to altered lipids.

A lipid pathway enrichment analysis (LIPEA) was performed to link each identified lipid to relevant metabolic or signaling pathways (Figure 16A). Among the top enriched pathways were glycerophospholipid and sphingolipid metabolism, arachidonic acid and cholesterol metabolism, inositol phosphate, bile secretion, steroid biosynthesis, and advanced glycation end (AGE) products/receptor of AGE (RAGE) signaling. Inflammatory and vascular pathways, including vascular smooth muscle cell contraction and TRP channel regulation, were also identified. Additional enriched pathways involved ferroptosis, autophagy, and key signaling cascades (e.g., PI3K/AKT, TNF, AMPK, and mTOR), underscoring adiposome lipids’ roles in immune, vascular, and metabolic regulation. KEGG mapping highlighted significant lipid perturbations in cholesterol metabolism, prostaglandin, and leukotriene pathways, eicosanoid synthesis, and lipid biosynthesis (Figure 16B). Overall, these findings indicate a distinct clustering of lipid alterations concentrated in inflammation, energy metabolism, and membrane homeostasis pathways.

## Discussion

Adiposomes are increasingly recognized as key mediators of interorgan communication, and we have previously reported their role in signaling pathways between adipocytes and vascular cells (17, 18, 22, 23). While the protein and nucleic acid content of EVs, including adiposomes, has been extensively studied, their lipid composition has received less attention, particularly in the context of cardiometabolic health. To address this gap, we performed comprehensive lipidomics profiling of adiposomes isolated from the VAT of participants with obesity and those who are lean, alongside a detailed assessment of cardiometabolic risk factors. First, we observed that the concentration of adiposomes was nearly twice as high in samples from patients with obesity compared with those from lean controls, corroborating findings from prior studies by our group and others (17, 19, 24). Such elevated EV release may modify paracrine and endocrine signaling, potentially contributing to metabolic dysregulation. This study suggests that the adiposome lipidomic landscape could be a biomarker or mediator of obesity-related inflammation and insulin resistance.

Our comprehensive lipidomics analysis identified 266 lipid species that differed significantly between obese and lean adiposomes. In particular, the PC, LPC, and other phospholipids (PE, ether-linked PE [EtherPE], EtherPC) were significantly lower in individuals with obesity. These findings align with previous studies that reported similar reductions in PC/LPC species in the plasma of individuals with obesity with or without diabetes compared with lean controls (25–27). Phospholipids are crucial for membrane integrity, cellular signaling, and lipid transport, and their depletion may compromise adiposome stability or alter their biological interactions (28). Phospholipid insufficiency has also been linked with impaired insulin signaling and a higher risk of cardiovascular disease, possibly due to compromised membrane fluidity and diminished capacity for NO production (29–34). Indeed, our investigation revealed a significant inverse relationship between total and individual phospholipid levels and BMI, VAT mass, fasting plasma insulin, HOMA-IR, and circulating leptin concentrations. Conversely, a positive correlation was identified between adiposome phospholipid levels and circulating NO, brachial FMD, and arteriolar FID.

Elevated Cer levels, particularly those with moderate chain lengths and saturated FA, were a hallmark of obese adiposomes, correlating with adiposity, insulin resistance, systemic inflammation, and vascular dysfunction. Obesity-driven sphingolipid metabolism shifts toward Cer accumulation, often depleting SM (35). Indeed, we observed lower SM levels, suggesting upregulated sphingomyelinase (SMase) activity converting SM into Cer. Ceramides are linked to insulin resistance, inflammation, and endothelial dysfunction by disrupting insulin receptor signaling and promoting mitochondrial reactive oxygen species (36). They also trigger proinflammatory cascades, worsening metabolic and vascular dysfunction (37). Our findings align with studies showing elevated plasma Cer in obesity, insulin resistance, and diabetes (38). However, this study identifies ceramide enrichment specifically within adiposomes, highlighting their potential role in mediating obesity-related metabolic and vascular complications. Several studies have previously reported that acid SMase (aSMase) and neutral SMase (nSMase) are elevated in adipose tissue from obese versus lean groups, resulting in increased ceramide generation, inflammation, and insulin resistance. These enzymatic alterations have been linked to impaired lipid metabolism and cardiometabolic diseases (39–41). These findings highlight the importance of SMase activity, suggesting that future studies measuring aSMase and nSMase activities could provide valuable insights into their role in shaping the lipid profiles identified in this study.

Notably, Cer in lean individuals were predominantly longer (≥ 38 carbons) and/or unsaturated, such as Cer 42:0, Cer 43:2, and Cer 26:2, while those in individuals with obesity were shorter (≤ 26 carbons) and/or saturated, such as Cer 18:0/22:0 and Cer 15:1/15:0. Prior studies link plasma Cer with C14–C26 acyl chains to obesity, metabolic disorders, and vascular dysfunction, whereas ultra–long chain Cer (>26 carbons) are essential for cellular integrity and skin barrier function (42, 43). C16 and C18 ceramides are particularly associated with obesity, type 2 diabetes, and liver steatosis, with studies showing that disrupting C18 Cer synthesis improves insulin sensitivity and glucose tolerance in high-fat diet models (44, 45). The mechanism behind the abundance of short unsaturated Cer is not known. However, some plausible explanations for this phenomenon could be related to the increased activity of the enzymes involved in the initial steps of Cer synthesis, such as serine palmitoyltransferase, favoring the incorporation of saturated FAs (like palmitate) into Cer backbones. Additionally, metabolic overload may impair FA elongation and desaturation, leading to an imbalance favoring short-chain saturated Cer (46). Further studies are needed to elucidate these mechanisms.

Our previous studies show that adiposomes from individuals with obesity induce structural and functional changes in endothelial cells, including caveolae disruption, impaired endothelial NO synthase (eNOS) signaling, and compromised membrane integrity (17, 18, 22, 23). Building on this, our work links dysregulated adiposome Cer to vascular dysfunction, marked by reduced brachial artery dilation, impaired arteriolar vasoreactivity, elevated systolic blood pressure, and lower NO bioavailability. Additionally, adiposome Cer levels strongly correlated with circulating inflammatory markers CRP and IL-6, highlighting their role in systemic inflammation and vascular impairment.

Adiposomes from the obese group also displayed higher free FAs and ACar. Elevated ACar can reflect incomplete mitochondrial FA oxidation, contributing to lipotoxic intermediates that worsen insulin sensitivity and prompt inflammatory pathways (47). Conversely, ACar accumulation could be interpreted as an indicator of increased FA flux. Future functional studies are required to directly investigate the role of ACar-rich adiposomes in modulating mitochondrial oxidation capacity, providing a more mechanistic understanding of their effect on metabolic regulation (48, 49). Similarly, excess-free FAs from dysfunctional adipocytes exacerbate ectopic fat deposition, insulin resistance, and pancreatic β cell dysfunction (50). In this study, excess FAs and ACar correlated positively with BMI, higher fat mass, insulin resistance, systemic inflammation, and vascular dysfunction, reinforcing their potential role as markers or mediators of adiposity-driven metabolic stress. Conversely, lipids known for their insulin-sensitizing and antiinflammatory properties, such as FAHFAs, were significantly lower in individuals with obesity. Overall, these lipidomic changes converge into a state that promotes metabolic stress and vasculopathy, underlining the possibility that adiposomes are conduits through which pathological lipids traffic between local adipose depots and systemic circulation.

Examining the link between adiposome lipidomic alterations and metabolic disease states, individuals with obesity who have diabetes or insulin resistance exhibited higher ACar and lower phospholipids and FAHFAs compared with those who have obesity but are metabolically healthy. This pattern aligns with studies associating impaired mitochondrial FA oxidation and FAHFA depletion with poorer glycemic control (51, 52). Similarly, hypertensive individuals with obesity exhibited elevated Cer and reduced SM, suggesting an upregulated SMase pathway that not only yields Cer but may directly modulate vascular reactivity. Significant alterations were observed in adiposome Cer, DG, and specific free FAs among individuals with hepatic steatosis. This highlights the potential role of adiposomes in the interplay between adipose tissue and the liver in obesity, where excessive lipolysis and free FA release may contribute to hepatic lipid accumulation and the development of fatty liver (53). Regression analyses in the current study further support these relationships between adiposome lipids and metabolic diseases/risks, as revealed by the strong relationships between adiposome lipids and cardiometabolic indices, including BMI, WC, HOMA-IR, systemic inflammation, and vascular function (brachial FMD and arteriolar FID). Ceramides, free FAs, and ACar were positively correlated with adiposity and insulin resistance but were inversely related to endothelial function. At the same time, SM and FAHFAs displayed protective associations linked to improved glycemic control and vascular health. Prior research has linked circulating lipids such as monoacylglycerols, DG, free FAs, and cholesteryl esters to cardiometabolic risk, emphasizing the roles of specific lipids in obesity, insulin resistance, inflammation, and vascular health (54). However, our study investigates these relationships within adiposomes, offering comprehensive insights into their contribution to metabolic and vascular dysfunction.

Obese adiposomes had lower TG levels, regardless of whether they were on lipid-lowering medication, which may indicate reduced lipogenesis or increased lipolysis. In obesity, lower lipogenesis might reflect an impaired ability to synthesize and store TGs due to associated metabolic stress or hormonal imbalances (55), while enhanced lipolysis may suggest an increased breakdown of stored TGs driven by elevated adipose tissue inflammation, insulin resistance, or heightened catecholamine activity (56–58). Higher adiposome TG levels were also observed in hypertensive compared with nonhypertensive individuals with obesity, potentially influenced by antihypertensive medications, particularly calcium channel blockers (e.g., amlodipine) and β blockers (59). These drugs can alter lipid metabolism by affecting adipocyte hormonal and signaling pathways. Our analysis confirmed a significant effect of these medications on adiposome TG and SM levels. To better understand the paradox of having lower TG in obese adiposomes, future studies should include comprehensive analyses of TG content in the corresponding adipose tissue, as well as the activity and expression levels of key enzymes in lipid metabolism, such as lipoprotein lipase, hormone-sensitive lipase, or adipose TG lipase. Such studies may provide valuable insights into the role of adiposome lipids in obesity and related metabolic disorders.

One of the unique aspects of this study was applying machine learning algorithms to classify obesity, cardiometabolic diseases, and clinically relevant risk states (vascular dysfunction, systemic inflammation) using adiposome lipidomic profiles. Our models accurately distinguished individuals with obesity from those who are lean, often surpassing 90% accuracy with strong sensitivity and specificity. Notably, certain Cer species (Cer-NS d15:1/15:0, Cer-NS d18:1/23:0) and FAHFAs (FAHFA 18:0/20:2) emerged repeatedly among the top discriminators, underscoring their utility as robust biomarkers. These models were similarly effective at stratifying participants with obesity who have diabetes, hypertension, dyslipidemia, and liver steatosis versus those who do not. Although the random forest models tended to have slightly lower sensitivity in some comparisons (e.g., ~63% for diabetic versus nondiabetic classification), the decision trees often performed exceptionally well (up to ~94% accuracy for diabetic and ~91% for hypertensive classification). These data collectively argue that adiposome lipid profiles could help identify individuals at the greatest risk of obesity-related complications, informing targeted intervention strategies.

Our machine learning analyses classified participants as having normal or impaired vascular function based on brachial FMD or arteriolar FID thresholds and low or high inflammation defined by IL-6 and CRP levels. Classification accuracy often exceeded 85% and sometimes reached 98%–100% in the impaired vascular function group, underscoring the strong predictive power of adiposome lipid composition. Similarly, individuals with high inflammation were identified with high accuracy. Notably, Cer-NS d15:1/15:0 repeatedly emerged among the top features, suggesting a key role in endothelial dysfunction and inflammatory signaling. These findings highlight the translational potential of adiposome lipidomics. With standardized protocols and larger validation cohorts, it may be possible to develop clinically validated models to predict or monitor insulin resistance, vascular dysfunction, and inflammation in obesity, enabling personalized therapies and earlier interventions.

Finally, our LIPEA/KEGG-based pathway enrichment results revealed perturbations in glycerophospholipid, sphingolipid, and FA metabolism, along with pathways including arachidonic acid, inositol phosphate, and steroid biosynthesis, among others. Notably, the involvement of AGE/RAGE signaling and vascular smooth muscle contraction hints that these lipidomic changes may converge on pathways known to modulate endothelial homeostasis and vasoreactivity. Moreover, detecting ferroptosis, autophagy, and central signaling cascades (TNF, PI3K/Akt, mTOR, AMPK) underscores the broad cellular ramifications of adiposome lipids. Ceramides, for instance, are recognized triggers of autophagy and apoptosis in endothelial cells, while specific phospholipids might influence ferroptosis by modulating lipid peroxidation (60–62). This network-level view supports a scenario in which adiposome-mediated lipid transport from hypertrophic or inflamed adipose tissue exerts far-reaching systemic effects, orchestrating multiple mechanistic arms of metabolic and vascular pathology.

In summary, adiposomes loaded with bioactive lipids may carry signals from dysfunctional adipose tissue to distant organs. Elevated Cer and free FAs in these vesicles can drive ectopic fat deposition, inflammatory cytokine release, and disrupted insulin signaling, while the depletion of protective lipids (FAHFAs, phospholipids, SM) dampens antiinflammatory capacity (63, 64). Over time, this imbalance can lead to chronic inflammation, hepatic steatosis, and vascular remodeling that promotes hypertension and atherosclerosis. Although it remains unclear whether adiposome changes drive or simply reflect metabolic decline, our data suggest that adiposomes act as integrative agents in obesity pathophysiology. These findings align with growing evidence that obesity increases EV secretion of proinflammatory, insulin-desensitizing cargo. By providing a high-resolution lipidomic profile in participants who are lean or have obesity, we extend these observations and show that machine learning classifiers can accurately distinguish multiple metabolic phenotypes from adiposome lipid patterns. Unlike many studies on plasma-derived EVs, we examined adiposomes directly from adipose tissue biopsies, giving a tissue-specific view of vesicle composition. Strong links between adiposome lipids, cardiometabolic indices, and vascular function underscore a pivotal role for local adipose-derived vesicles in obesity, expanding the concept of adipo-organ crosstalk.

Adiposomes carry diverse lipids reflective of adipose tissue function and metabolic states. They uniquely contain high neutral lipid levels, notably TG, DG, and CE, aligning with adipocytes’ role in lipid storage. Adiposomes are also rich in phospholipids like PC, PE, and PS, with distinct fatty acyl chain profiles compared with EVs from other cells. Additionally, bioactive lipids such as FA, FAHFA, Cer, and SM highlight adiposomes’ roles in metabolic regulation and inflammation (63, 65). EV lipidomes differ by cellular origin: MSC-derived EVs contain lower neutral lipids but higher cholesterol, PS, and Cer (66). Hepatocyte-derived EVs emphasize phospholipids, complex cholesterol metabolites, and higher sphingolipids (67), while neuronal-derived EVs feature glycolipids like gangliosides, high cholesterol, and SM levels but lower neutral lipids (68). Platelet and plasma EVs are enriched in PS, essential for coagulation, yet show lower neutral lipids compared with adiposomes (69). Muscle-derived EVs transport unique Cer and glycerolipids, affecting adipocyte function (70). Future studies will systematically compare our adiposome lipidome with datasets from various tissues available in repositories like Vesiclepedia (http://www.exocarta.org/) and ExoCarta (http://www.microvesicles.org/) to identify unique and shared lipid signatures across sources. Due to their complexity, such evaluations extend beyond this study but remain essential to our future research.

This study has many strengths, including (a) comprehensive phenotyping, for which we integrated extensive clinical, biochemical, vascular, and lipidomic data, enabling robust correlations and machine learning analyses. (b) For high-resolution lipidomics, our approach encompassed 595 lipid species spanning 19 classes, capturing a broad scope of lipid perturbations. (c) Direct adiposome isolation used adipose tissue biopsies to reduce confounding from EVs of nonadipose origin and confirm adipocyte-specific markers (PPARγ, FABP4). (d) Comparisons across comorbidities categorized participants with obesity by diabetes, hypertension, dyslipidemia, and liver steatosis status, providing valuable insight into differential lipid signatures linked to each condition. However, this study has limitations, including (a) cross-sectional design, since our findings reveal associations rather than causal relationships. A longitudinal approach could clarify whether the lipid shifts precede or follow the onset of insulin resistance and vascular dysfunction. (b) Sample size and diversity is another limitation. Although the total sample was sizable (*n* = 122), subgroups with particular conditions (e.g., dyslipidemic versus nondyslipidemic) were smaller, potentially limiting statistical power for certain comparisons. More ethnically and geographically diverse cohorts are also needed to generalize the results. (c) Medication effects is a limitation because many participants with obesity were on antihypertensive, antidiabetic, or lipid-lowering therapy. While our analyses tracked and accounted for these medications, isolating their full effect on lipidomic profiles remains challenging. (d) Functional studies is a limitation because, although we identified potential mechanistic pathways, our data remains largely correlative. Future functional experiments (e.g., adiposome transfer to cultured cells or by injecting animals) are needed to confirm the direct biological actions of specific lipid species. (e) While optimized to broadly detect multiple lipid classes, our untargeted lipidomics approach had limited sensitivity for complex sphingolipids such as gangliosides. Therefore, efforts are currently underway to optimize a targeted sphingolipidomic method to comprehensively assess these species in future studies. (f) The current analysis utilized 27 internal standards, ensuring that at least 2 distinct standards represented each lipid class. Five of these standards showed statistically significant differences between the lean and obese groups. These changes were likely due to matrix effects, such as ion suppression or coelution with endogenous lipids (71). To maintain reliable quantification, we excluded these standards from further analysis, ensuring the stability of our results and minimizing the risk of skewed data. (g) Finally, we did not include an overweight category in our analysis. Including this group would have provided a more nuanced understanding of how lipid profiles and adiposome content change across the full spectrum of body weight. Despite these limitations, the study offers a compelling portrait of how adiposome lipid composition is reshaped in obesity, pointing to potential diagnostic and therapeutic strategies in obesity-related diseases.

### Conclusion and future perspectives.

In summary, this study demonstrates that obesity is linked with pronounced alterations in adiposome lipid content, including elevated Cer, free FAs, and ACar and reduced phospholipids, SM, and FAHFAs. These disruptions correlate strongly with systemic inflammation, insulin resistance, and endothelial dysfunction, affecting adiposomes as active participants in obesity pathogenesis rather than mere bystanders. However, while these findings illustrate robust associations, they do not establish causation. By leveraging advanced lipidomics and machine learning, we identified lipid species and overall lipid classes that not only distinguish individuals with obesity from those who are lean but also accurately classify comorbidities (diabetes, hypertension, dyslipidemia, steatosis) and predict impaired vascular function or high inflammation states. Our work underscores the potential utility of adiposome-derived lipid signatures in improving risk assessment, early diagnosis, and eventually personalizing therapies for obesity-related metabolic and cardiovascular diseases. Future studies aimed at mechanistically unraveling how adiposomes orchestrate intertissue communication, validating candidate lipids as functional mediators, and exploring interventions that selectively modify adiposome cargo hold promise for advancing obesity management. The comprehensive view provided here enriches our understanding of the lipid-mediated crosstalk in obesity and sets the stage for translational research targeting EV biology for cardiometabolic benefit.

Future work should prioritize mechanistic validation and causality. In vitro exposures of endothelial cells or hepatocytes to purified adiposomes with specific lipid profiles could determine whether Cer, free FAs, or depleted FAHFAs directly impair insulin signaling, activate inflammatory pathways, or compromise NO-mediated vasodilation. Animal models of diet-induced obesity manipulating adiposome production or lipid species would reveal if reversing specific lipid imbalances improves metabolic and vascular outcomes. Another promising avenue involves targeting adiposome secretion or cargo. Small-molecule inhibitors of nSMase may reduce Cer overproduction in adiposomes, enhancing insulin sensitivity and vascular function. Conversely, boosting protective lipids like FAHFAs could restore immunometabolic balance. Such interventions demand a thorough understanding of adipocyte-derived EV biogenesis and downstream molecular targets. Moreover, our machine-learning data suggest that analyzing adiposome lipidomic signatures could guide personalized risk assessments. Moreover, integrating lipidomics with proteomics or metabolomics may refine biomarker accuracy and uncover additional mechanistic insights for obesity management.

Studies comparing lipids in EV membranes versus their internal contents highlight distinct lipid profiles, with EV membranes being enriched in cholesterol, SM, and PC. These lipids contribute to enhanced membrane stability, rigidity, and cargo sorting. In contrast, the internal lipid content, including TGs and cholesteryl esters, reflects cellular metabolic states or stress (72). The interplay between membrane and internal lipids is crucial for regulating EV function. While the current investigation did not differentiate between membrane and internal EV lipids, our future studies will specifically address this distinction to gain deeper insights into how these lipid profiles govern EV biogenesis, cargo composition, and their role in intercellular communication, which could be pivotal for advancing therapeutic applications targeting EVs. Finally, future studies are required to compare the lipidome of s.c. and visceral adiposomes to advance our understanding of how distinct adipose depots influence vascular and metabolic health.

## Methods

### Sex as a biological variable.

In designing this cross-sectional study, we recruited both male and female adults undergoing elective surgery, ensuring adequate representation in lean and obese groups. Clinical and laboratory assessments (e.g., metabolic and vascular parameters) were recorded separately for men and women. Data were analyzed for main effects of sex and potential interactions with adiposity. Where applicable, post hoc analyses explored sex-specific differences in adiposome release, lipid composition, and associated cardiometabolic outcomes.

### Subject enrollment.

This study recruited 75 adults with obesity (45 females, 30 males, BMI ≥ 30 kg/m^2^) undergoing bariatric surgery (sleeve gastrectomy) and 47 lean adults (26 females, 21 males, BMI < 25 kg/m^2^) undergoing elective surgery (e.g., hernia repair) at the University of Illinois Hospital in Chicago. The race/ethnicity composition of the obese group included AA (*n* = 42), H (*n* = 20), and NHW (*n* = 13). In the lean group, the distribution was as follows: AA (*n* = 23), H (*n* = 15), and NHW (*n* = 9). Sex and race/ethnicity were self-reported. Ages ranged from 19 to 50 years. Blood samples and physiological measurements were taken 2–3 weeks before surgery, and VAT was collected during surgery. Exclusion criteria included current pregnancy, smoking, previous bariatric surgery, and current liver failure, renal failure, heart failure, cancer, or autoimmune disease.

### Cardiometabolic risk, adiposome isolation and lipid analysis, and vascular function assessment.

Detailed methods are provided in the Supplemental Methods.

### Statistics.

All statistical analyses were conducted using SPSS statistical software (version 26.0; SPSS Inc.) and RStudio (v.4.4.1). Data are presented as the mean ± SD for continuous variables or frequencies and percentages for categorical variables. All variables were tested for normality using the Shapiro-Wilk test and visualized using histograms and Q-Q plots. Based on the results, parametric tests were used for normally distributed data, while nonparametric tests were applied for nonnormally distributed data. 2-tailed Student’s *t* tests or 1-way ANOVA, adjusted for age, sex, and race/ethnicity, assessed significance, and *P* values were corrected by Benjamini-Hochberg FDR. When the assumptions of the *t* test were not met, the Mann-Whitney *U* test was used. Log_2_ FC was calculated as the log_2_ of the ratio of average expression in the obese group to that in lean controls. Linear regression models (adjusted for age, sex, and race/ethnicity) evaluated associations between lipids and BMI. The reported estimates (β) represent the effect of a 1-SD increase in the independent variables.

PCA was conducted using RStudio on scaled data (mean 0, SD 1) via the prcomp function that uses singular value decomposition (SVD). Hierarchical clustering was performed using the Ward method on scaled data, and data matrices were visualized using the heatmap function to identify clusters. Visualizations (bar plots, box plots, volcano plots) were generated with ggplot2. A statistically significant linear relationship between continuous variables was tested using a bivariate Pearson or Spearman correlation. Lipids with FDR < 0.05 were imported into LIPEA for pathway mapping using Fisher’s exact tests.

IBM SPSS (version 29) was employed to produce both random forest plots and decision trees and to configure prediction models. Obesity, diabetes, hypertension, dyslipidemia, liver steatosis, vascular dysfunction, and high systemic inflammation were used as the response variables, and adiposome concentrations of lipid species were used as quantitative exploratory variables. For random forest, the sample size was 122 for predicting obesity and 75 for predicting cardiometabolic diseases in the obese group; the number of trees ranged from 300 to 500, the maximum tree depth was 20, and imbalanced data were handled by adjusting weights inversely proportional to class frequencies. Decision trees were constructed via the Classification and Regression Trees (CART) algorithm included in SPSS’s Decision Trees module, using a minimum parent size of 5, a minimum son size of 2, a maximum tree depth of 3, and a Gini splitting criterion. The model was pruned and validated with standard cross-validation (10-fold) to minimize overfitting. Each prediction model’s performance was then assessed using metrics such as accuracy, sensitivity, specificity, and the AUC of the ROC, ensuring interpretable results.

### Study approval.

The research was performed in compliance with the Declaration of Helsinki and approved by the IRB of the University of Illinois at Chicago (protocol no. 2021-1113, approved in October 2021). Informed written consent was obtained from all the participants involved in the study.

### Data availability.

The datasets used and/or analyzed during the current study are available in the Supporting Data Values file and can also be obtained from the corresponding author upon reasonable request.

## Author contributions

EM, IM, AM, MMA, MCA, and AMM generated data and figures. IM, MHM, GS, FMB, MAM, and CH assisted with patient recruitment and sample collection. BTL and AMM led study conceptualization, manuscript review, and editing. AMM secured funding and supervised the research. All authors reviewed and approved the final manuscript.

## Supplementary Material

Supplemental data

ICMJE disclosure forms

Unedited blot and gel images

Supporting data values

## Figures and Tables

**Table 1:**
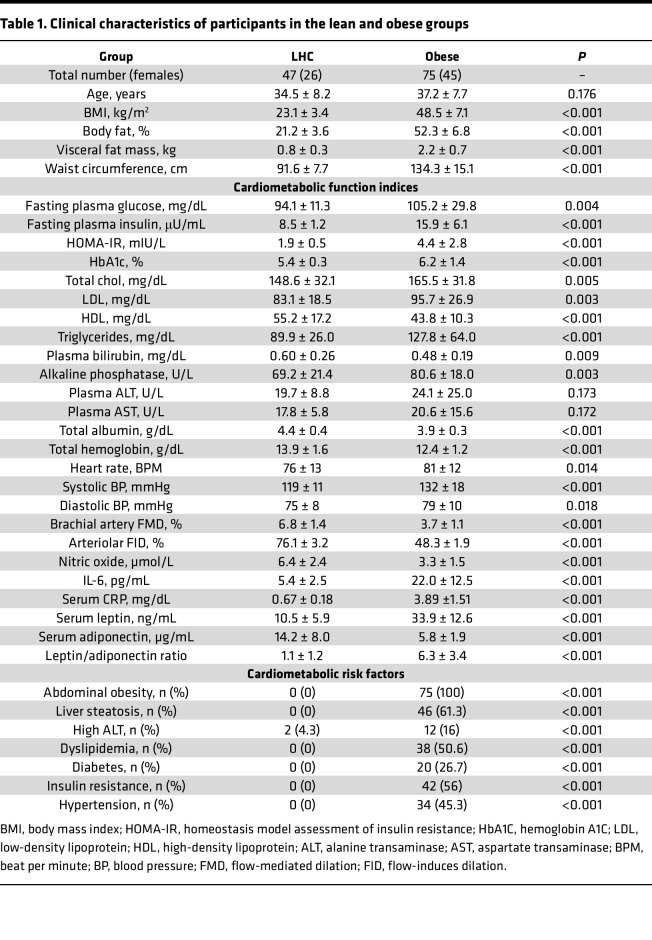
Clinical characteristics of participants in the lean and obese groups

**Table 2:**
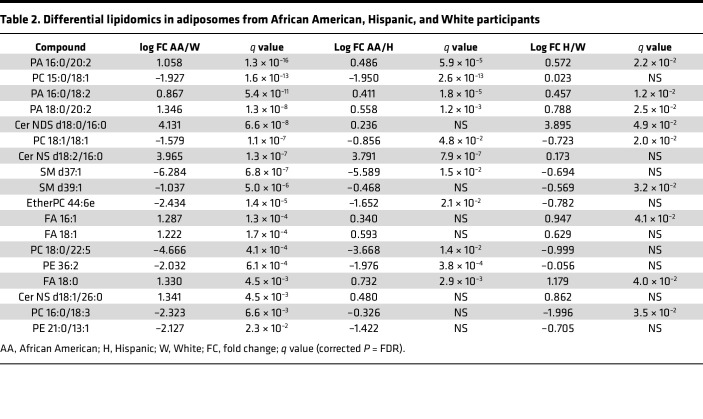
Differential lipidomics in adiposomes from African American, Hispanic, and White participants

## Version 1 06/23/2025

Electronic publication
