# Supplementary material for: Lipidomic profiling of human adiposomes identifies specific lipid shifts linked to obesity and cardiometabolic risk
Source: JCI Insight. 2025 Jun 23;10(12):e191872. doi: 10.1172/jci.insight.191872 (PMC12226050; doi:10.1172/jci.insight.191872)
Supplement: Supplemental data [file jciinsight-10-191872-s040.pdf]

Methods

Cardiometabolic risk measurements

Anthropometric data (body weight, BMI) were recorded, and body composition (fat versus lean mass), was quantified using DEXA (iDXA, General Electric Inc). Fasting glucose, insulin, and Hemoglobin A1c were quantified using established protocols [1], and insulin sensitivity was estimated by the homeostasis model assessment of insulin resistance (HOMA-IR), calculated as fasting insulin (U/L) × fasting glucose (nmol/L)/22.5[2]. Serum lipids (total cholesterol, LDL, HDL, TGs) were measured using enzymatic assays[1, 3, 4] (Roche Diagnostics, Indianapolis, IN, USA). Routine liver function tests (ALT, AST, bilirubin, albumin, alkaline phosphatase) were conducted in the hospital laboratory. Plasma NO was determined via a nitrate/nitrite kit (Cayman Chemicals, Ann Arbor, MI)[5-7]. Circulating inflammatory biomarkers, IL6 and CRP were quantified via High Sensitivity immunoassays (R&D Systems, Minneapolis, MN, USA). Serum leptin and adiponectin were measured using Invitrogen™ Leptin (KAC228) and Adiponectin (KHP004) Human ELISA Kits (Invitrogen, Waltham, MA, USA). Liver steatosis was evaluated using the Aplio i-series liver Attenuation Imaging (ATI) package (Aplio i900; Canon Ultrasound Systems, Melville, NY, USA), whereby a low-frequency curvilinear transducer visualized the right hepatic lobe, and the ATI software calculated the attenuation coefficient (dB/cm/MHz) within regions of interest.

Adiposome isolation

Fat biopsies were prepared using the methods we published [8]. Briefly, the tissue was rinsed with sterile Medium 199 (Gibco, Waltham, MA, USA), cut into small fragments, and digested in a solution containing Type 1 collagenase (Worthington) and 4% bovine serum albumin (BSA) in Medium 199. After filtration and centrifugation at 500 g, the floating mature adipocytes were harvested and placed on inserts in Medium 199 supplemented with 1% penicillin/streptomycin and 5% exosome-free fetal bovine serum (FBS). After 24-48 hours, the conditioned medium was centrifuged at 1,000 g (5 minutes), then 15,000 g (15 minutes), filtered (0.45 μm), and ultracentrifuged at 150,000 g for 2 hours to pellet adiposomes, which were then resuspended and analyzed with a Nanoparticle Analyzer (NanoSight NS300, Malvern Instruments Ltd, Malvern, UK). For TEM visualization, adiposomes were fixed in 2% paraformaldehyde, placed on formvar/carbon film-coated 200-mesh copper EM grids, stained with filtered uranyl acetate, and examined using a JEM-3010 TEM; JEOL USA, Inc., Peabody, MA, USA). Proteins were extracted from adiposomes using RIPA lysis buffer, quantified with the ThermoFisher

Scientific Pierce BCA Protein Assay, separated on 4%–12% Bis-Tris gradient gels, and transferred onto a polyvinylidene fluoride membrane. Membranes were incubated overnight with primary antibodies against CD9, CD81, CD63, PPARγ, adiponectin, apolipoprotein B (APOB), and FABP4 (Cell Signaling, Danvers, MA, USA) followed by a 1-hour incubation with infrared (IRDye) secondary antibodies (LI-COR Biosciences) then dried and imaged using the Odyssey Clx infrared imaging system (700 nm for IRDye680TM and 800 nm for IRDye800TM).

Adiposome lipid extraction and analysis

Lipids were extracted from adiposomes via the Folch method[8] and analyzed on an Agilent 6545 Q-TOF LC-MS system using a 2.1 × 100 nm Agilent Poroshell C18 (2.7 μm) column (Agilent Technologies, Santa Clara, CA, USA) at a flow rate of 300 μL/min. The gradient started at 70% solvent B (0-1 min), ramped to 86% B (3.5-10 min), reached 100% B (11-17 min), and was re-equilibrated for 5 minutes. Key settings included 3000 V VCap, 145 V fragmentor, 12 L/min sheath gas at 350°C, and 11 L/min drying gas at 200°C. Lipid identification used Lipid Annotator software (Agilent), which generated an MS/MS library based on m/z precursors and retention times for each identified lipid. Profinder (vB.10.00, Agilent) then extracted molecular features for peaks ≥ 5000 counts with a quality score > 60, and the presence of at least two isotopes. Retention times were aligned to ±0.1 min within a mass accuracy window of ≤5.0 ppm, and peaks were integrated using the Agile integrator. Data were baseline-corrected, normalized to internal standards, and exported to Mass Profiler Professional exported to Mass Profiler Professional (v15.1, Agilent) for individual analysis.

Vascular function assessment

Brachial artery FMD was measured using a vascular probe (Aplio i900; Canon Ultrasound Systems, Melville, NY, USA), placing a blood pressure cuff on the forearm inflated to 220 mmHg for 5 minutes. The arterial diameter was recorded 1 minute before cuff inflation (baseline) and 5 minutes post-deflation (reactive hyperemia), with images analyzed using Automated Edge Detection software. FMD (%) was calculated by subtracting the baseline diameter from the peak hyperemia diameter, dividing by the baseline value, and multiplying by 100 [1, 3, 6, 7, 9]. For arteriolar FID, fat arterioles were dissected from adipose samples, cleaned of excess tissue, and cannulated in an organ chamber as previously described [4-7, 10, 11]. After being tied onto glass microcapillaries with nylon sutures, the arterioles were perfused with Krebs buffer under increasing pressure gradients (10-100 cm H_2O). Their intraluminal diameters were monitored via an inverted Olympus microscope. Baseline vasoconstriction was

induced with endothelin-1 (ET-1; 10^{-6} mol/L), and FID was quantified by normalizing the maximum arteriolar diameter at each pressure to the ET-1-constricted diameter.

1. Haloul M, Vinjamuri SJ, Naquiallah D, Mirza MI, Qureshi M, Hassan C, et al. Hyperhomocysteinemia and low folate and vitamin b12 are associated with vascular dysfunction and impaired nitric oxide sensitivity in morbidly obese patients. *Nutrients*. 2020;12
2. Matthews DR, Hosker JP, Rudenski AS, Naylor BA, Treacher DF, Turner RC. Homeostasis model assessment: Insulin resistance and beta-cell function from fasting plasma glucose and insulin concentrations in man. *Diabetologia*. 1985;28:412-419
3. Ali MM, Naquiallah D, Qureshi M, Mirza MI, Hassan C, Masrur M, et al. DNA methylation profile of genes involved in inflammation and autoimmunity correlates with vascular function in morbidly obese adults. *Epigenetics*. 2021:1-17
4. Ali MM, Hassan C, Masrur M, Bianco FM, Naquiallah D, Mirza I, et al. Adipose tissue hypoxia correlates with adipokine hypomethylation and vascular dysfunction. *Biomedicines*. 2021;9
5. Mahmoud AM, Szczurek MR, Blackburn BK, Mey JT, Chen Z, Robinson AT, et al. Hyperinsulinemia augments endothelin-1 protein expression and impairs vasodilation of human skeletal muscle arterioles. *Physiol Rep*. 2016;4
6. Mahmoud AM, Szczurek M, Hassan C, Masrur M, Gangemi A, Phillips SA. Vitamin d improves nitric oxide-dependent vasodilation in adipose tissue arterioles from bariatric surgery patients. *Nutrients*. 2019;11
7. Mahmoud AM, Hwang CL, Szczurek MR, Bian JT, Ranieri C, Gutterman DD, et al. Low-fat diet designed for weight loss but not weight maintenance improves nitric oxide-dependent arteriolar vasodilation in obese adults. *Nutrients*. 2019;11
8. Mirza I, Haloul M, Hassan C, Masrur M, Mostafa A, Bianco FM, et al. Adiposomes from obese-diabetic individuals promote endothelial dysfunction and loss of surface caveolae. *Cells*. 2023;12
9. Ali MM, Naquiallah D, Hassan C, Masrur M, Bianco FM, Frederick P, et al. Obesity-associated hypoxia contributes to aberrant methylation of genes implicated in inflammation and vascular function. *The FASEB Journal*. 2020;34:1-1
10. Ali MM, Naquiallah D, Qureshi M, Mirza MI, Hassan C, Masrur M, et al. DNA methylation profile of genes involved in inflammation and autoimmunity correlates with vascular function in morbidly obese adults. *Epigenetics*. 2022;17:93-109
11. Ali MM, Phillips SA, Mahmoud AM. Hif1alpha/tet1 pathway mediates hypoxia-induced adipocytokine promoter hypomethylation in human adipocytes. *Cells*. 2020;9

Supplementary Table 1. Associations of lipid species that increased in obese individuals with cardiometabolic risk

	Total ACar		Total Cer		Total DG		Total FA	
	r	*P value*	*r*	*P value*	*r*	*P value*	*r*	*P value*
BMI	.471**	2.25E-08	.247**	3.01E-03	.504**	1.63E-09	.685**	1.63E-18
Total fat	.481**	1.05E-08	.317**	1.91E-04	.578**	1.61E-12	.718**	6.44E-21
VAT mass	.431**	3.67E-07	.385**	5.84E-06	.499**	2.45E-09	.574**	2.30E-12
WC	.434**	2.56E-03	.368**	9.81E-03	.444**	2.03E-03	.693**	3.52E-07
HR	.232**	5.15E-03	0.066	2.35E-01	0.133	7.26E-02	0.081	1.87E-01
SBP	.181*	2.28E-02	.167*	3.31E-02	.188*	1.88E-02	.268**	1.43E-03
DBP	0.009	4.61E-01	0.138	6.52E-02	0.091	1.60E-01	0.119	9.56E-02
FPI	.457**	5.88E-08	.205*	1.18E-02	.308**	2.75E-04	.367**	1.61E-05
FPG	.360**	2.29E-05	0.086	1.72E-01	0.087	1.72E-01	0.055	2.73E-01
HOMA-IR	.476**	1.55E-08	.174*	2.77E-02	.234**	4.67E-03	.270**	1.34E-03
HbA1C	.425**	5.48E-07	-0.006	4.74E-01	.313**	2.21E-04	.301**	3.74E-04
Total Cholesterol	0.091	1.60E-01	0.073	2.12E-01	.248**	2.97E-03	0.133	7.24E-02
LDL	0.068	2.28E-01	0.088	1.68E-01	.263**	1.69E-03	.167*	3.33E-02
HDL	-.375**	1.02E-05	-0.144	5.62E-02	-.268**	1.43E-03	-.324**	1.36E-04
Triglycerides	.190*	1.83E-02	0.094	1.52E-01	.271**	1.25E-03	.161*	3.86E-02
Nitric oxide	-.351**	3.66E-05	-.228**	5.85E-03	-.462**	4.14E-08	-.511**	9.44E-10
Total bilirubin	-.171*	3.00E-02	-0.065	2.37E-01	-.273**	1.19E-03	-.153*	4.61E-02
Alkaline phosphatase	0.128	8.05E-02	0.045	3.12E-01	.207*	1.12E-02	.229**	5.51E-03
AST	0.056	2.71E-01	-0.004	4.81E-01	.187*	2.00E-02	0.121	9.39E-02
ALT	0.006	4.74E-01	0.030	3.74E-01	.208*	1.14E-02	0.082	1.85E-01
albumin	-.184*	2.13E-02	-0.024	3.97E-01	-.222**	6.97E-03	-.306**	3.08E-04
Hemoglobin	-.285**	7.26E-04	-.194*	1.61E-02	-.352**	3.51E-05	-.313**	2.19E-04
FMD	-.362**	2.04E-05	-0.109	1.17E-01	-.435**	2.83E-07	-.568**	4.55E-12
Arteriolar FID at Δ 60	-.553**	2.04E-11	-.358**	2.52E-05	-.633**	2.76E-15	-.762**	1.15E-24
Leptin	.356**	2.91E-05	.216**	8.41E-03	.450**	1.01E-07	.530**	1.71E-10
Adiponectin	-.325**	1.28E-04	-.245**	3.31E-03	-.409**	1.41E-06	-.456**	6.44E-08
leptin/adiponectin ratio	.341**	6.04E-05	.201*	1.32E-02	.435**	2.71E-07	.437**	2.42E-07
IL6	.313**	2.23E-04	.304**	3.29E-04	.303**	3.50E-04	.489**	5.47E-09
CRP	.427**	4.71E-07	.253**	2.44E-03	.459**	5.43E-08	.536**	9.95E-11

ACar, acylcarnitine; Cer, ceramide; DG, diglycerides; FA, fatty acids; BMI, body mass index; VAT, Visceral adipose tissue; WC, waist circumference; HR, heart rate; SBP, systolic blood pressure; DBP, diastolic blood pressure; FPI, fasting plasma insulin; FPG, fasting plasma glucose; HOMA-IR, homeostasis model assessment of insulin resistance; HbA1C, hemoglobin A1C; LDL, low-density lipoprotein; HDL, high-density lipoprotein; ALT, alanine transaminase; AST, aspartate transaminase; FMD, flow-mediated dilation; FID, flow-induces dilation; IL6, interleukin 6; CRP, C-reactive protein.

Supplementary Table 2. Associations of lipid species that decreased in obese individuals with cardiometabolic risk

	Total EtherPC		Total FAHFA		Total PC/LPC		Total SM		Total TG	
	r	*P value*	*r*	*P value*	*r*	*P value*	*r*	*P value*	*r*	*P value*
BMI	-.384**	6.15E-06	-.594**	2.81E-13	-.545**	4.12E-11	-.154*	4.48E-02	-.502**	1.93E-09
Total fat	-.373**	1.16E-05	-.634**	2.43E-15	-.568**	4.48E-12	-0.141	6.11E-02	-.511**	8.87E-10
VAT mass	-.312**	2.29E-04	-.522**	3.62E-10	-.441**	1.79E-07	-0.048	3.01E-01	-.386**	5.54E-06
WC	-0.121	2.28E-01	-.591**	2.97E-05	-.315*	2.39E-02	-0.080	3.11E-01	-.490**	6.62E-04
HR	-.180*	2.33E-02	-0.138	6.43E-02	-.224**	6.51E-03	-.176*	2.64E-02	-.188*	1.89E-02
SBP	-.174*	2.73E-02	-.272**	1.21E-03	-.255**	2.33E-03	0.011	4.51E-01	-.189*	1.85E-02
DBP	-0.090	1.62E-01	-0.128	8.05E-02	-.188*	1.90E-02	-0.062	2.50E-01	-.168*	3.24E-02
FPI	-.344**	5.30E-05	-.413**	1.14E-06	-.456**	6.56E-08	-.198*	1.45E-02	-.361**	2.25E-05
FPG	-0.089	1.66E-01	-0.116	1.03E-01	-0.147	5.26E-02	-0.102	1.31E-01	-0.103	1.30E-01
HOMA-IR	-.286**	6.97E-04	-.336**	7.88E-05	-.354**	3.20E-05	-.168*	3.20E-02	-.283**	7.84E-04
HbA1C	-0.133	7.27E-02	-.243**	3.44E-03	-.227**	5.91E-03	0.031	3.66E-01	-.244**	3.37E-03
Cholesterol	-0.104	1.26E-01	0.001	4.97E-01	-0.138	6.44E-02	-0.050	2.91E-01	-0.146	5.41E-02
LDL	-.169*	3.16E-02	-.163*	3.68E-02	-0.073	2.13E-01	-0.029	3.75E-01	-0.133	7.16E-02
HDL	0.066	2.37E-01	.345**	5.07E-05	.183*	2.19E-02	-0.045	3.11E-01	.273**	1.19E-03
Triglyceride	-0.147	5.34E-02	-.207*	1.12E-02	-.245**	3.32E-03	-0.074	2.09E-01	-0.095	1.50E-01
Nitric oxide	.212**	9.65E-03	.383**	6.51E-06	.389**	4.68E-06	0.081	1.89E-01	.406**	1.72E-06
Total bilirubin	-0.006	4.74E-01	0.120	9.36E-02	0.061	2.53E-01	0.046	3.08E-01	-0.054	2.79E-01
Alkaline phosphatase	-0.110	1.15E-01	-.155*	4.44E-02	-.152*	4.71E-02	-0.092	1.56E-01	-0.127	8.19E-02
AST	-0.062	2.51E-01	-0.039	3.35E-01	0.050	2.92E-01	0.067	2.33E-01	0.031	3.66E-01
ALT	-0.014	4.38E-01	-0.032	3.63E-01	0.023	4.00E-01	0.064	2.44E-01	0.004	4.83E-01
Albumin	.233**	4.86E-03	.334**	8.56E-05	.299**	4.17E-04	0.124	8.60E-02	.414**	1.07E-06
Hemoglobin	.216**	8.48E-03	.286**	6.98E-04	.305**	3.25E-04	0.095	1.49E-01	.305**	3.22E-04
FMD	.375**	1.06E-05	.487**	6.20E-09	.557**	1.34E-11	.250**	2.70E-03	.645**	5.33E-16
Arteriolar FID at Δ 60	.362**	2.13E-05	.689**	8.41E-19	.571**	3.12E-12	0.090	1.61E-01	.547**	3.41E-11
Leptin	-.310**	2.52E-04	-.465**	3.32E-08	-.484**	7.96E-09	-0.130	7.72E-02	-.438**	2.21E-07
Adiponectin	0.148	5.17E-02	.400**	2.45E-06	.309**	2.70E-04	-0.055	2.72E-01	.230**	5.42E-03
leptin/adiponectin ratio	-.292**	5.49E-04	-.444**	1.46E-07	-.459**	5.14E-08	-0.023	4.02E-01	-.392**	3.89E-06
IL6	-.309**	2.73E-04	-.461**	4.70E-08	-.378**	8.82E-06	0.003	4.89E-01	-.385**	6.08E-06
CRP	-.405**	1.81E-06	-.566**	5.61E-12	-.538**	8.18E-11	-0.115	1.05E-01	-.503**	1.76E-09

PC, phosphatidylcholine; LPC, lysophosphatidylcholine; FAHFA, Fatty acyl esters of hydroxy fatty acid; SM, sphingomyelin; TG, triglycerides; BMI, body mass index; VAT, Visceral adipose tissue; WC, waist circumference; HR, heart rate; SBP, systolic blood pressure; DBP, diastolic blood pressure; FPI, fasting plasma insulin; FPG, fasting plasma glucose; HOMA-IR, homeostasis model assessment of insulin resistance; HbA1C, hemoglobin A1C; LDL, low-density lipoprotein; HDL, high-density lipoprotein; ALT, alanine transaminase; AST, aspartate transaminase; FMD, flow-mediated dilation; FID, flow-induces dilation; IL6, interleukin 6; CRP, C-reactive protein.
