# Supplementary material for: Lipidomic profiling of human adiposomes identifies specific lipid shifts linked to obesity and cardiometabolic risk
Source: JCI Insight. 2025 Jun 23;10(12):e191872. doi: 10.1172/jci.insight.191872 (PMC12226050; doi:10.1172/jci.insight.191872)
Supplement: Unedited blot and gel images [file jciinsight-10-191872-s042.pdf]

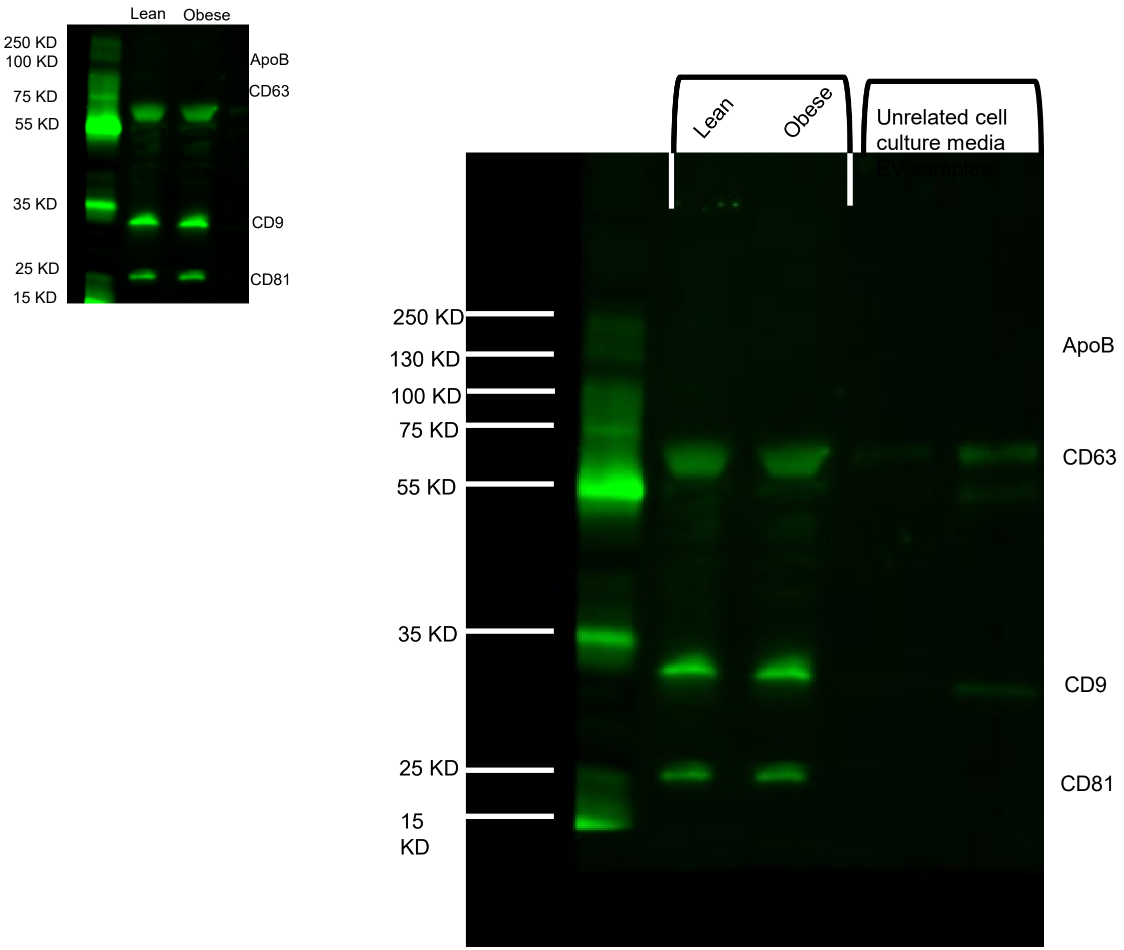

Fig.2E. Original Western blot of EV biomarkers (CD36, CD9, and CD81) and apolipoprotein B (ApoB) in two representative fresh samples.

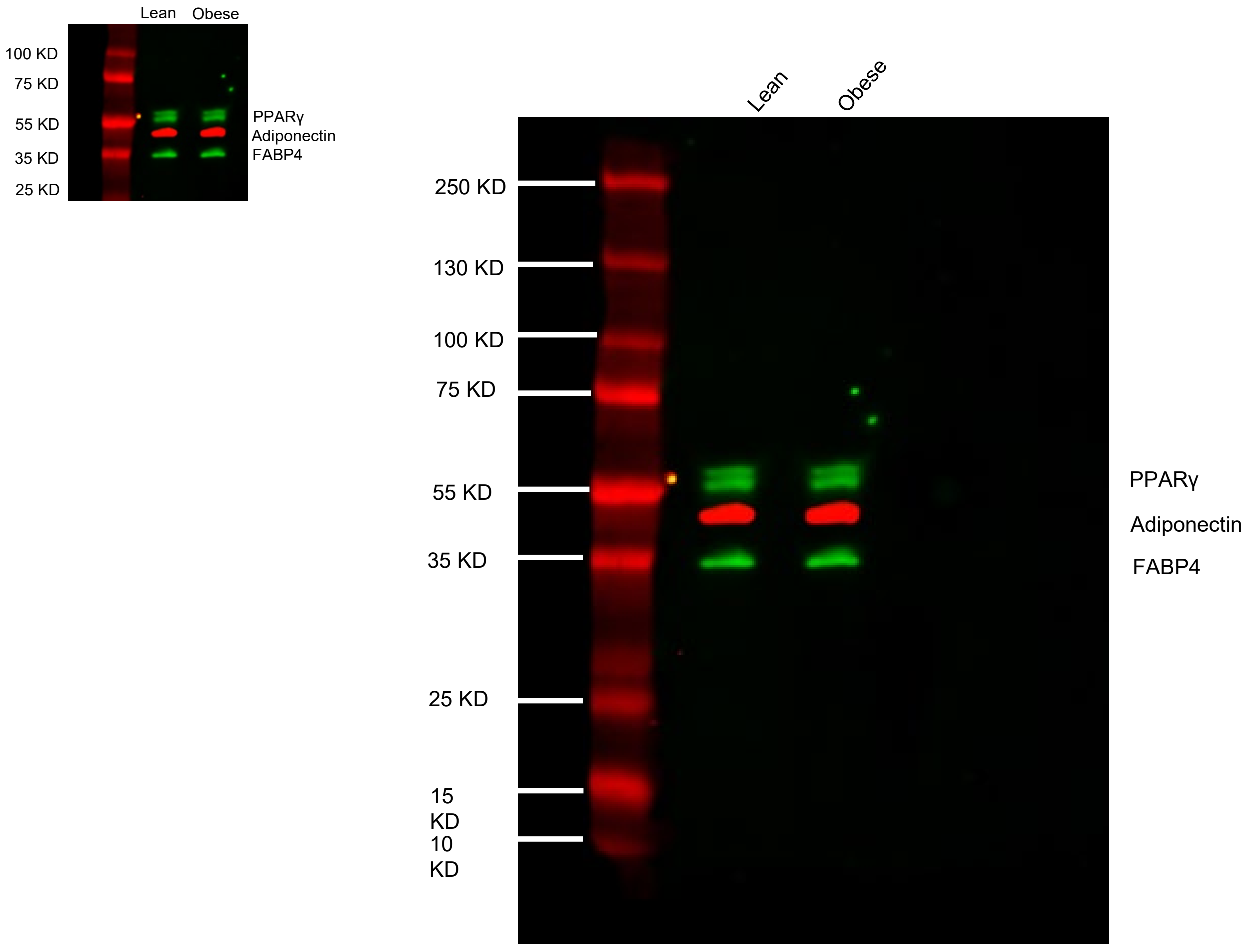

Fig.2F. Original Western blot of EV adipocytic proteins in two representative fresh samples (PPARγ, adiponectin, and FABP4).
